# MutViz 2.0: visual analysis of somatic mutations and the impact of mutational signatures on selected genomic regions

**DOI:** 10.1093/narcan/zcab012

**Published:** 2021-04-09

**Authors:** Andrea Gulino, Eirini Stamoulakatou, Rosario M Piro

**Affiliations:** Dipartimento di Elettronica, Informazione e Bioingegneria (DEIB), Politecnico di Milano, Via Ponzio 34/5, 20133 Milan, Italy; Dipartimento di Elettronica, Informazione e Bioingegneria (DEIB), Politecnico di Milano, Via Ponzio 34/5, 20133 Milan, Italy; Dipartimento di Elettronica, Informazione e Bioingegneria (DEIB), Politecnico di Milano, Via Ponzio 34/5, 20133 Milan, Italy

## Abstract

Patterns of somatic single nucleotide variants observed in human cancers vary widely between different tumor types. They depend not only on the activity of diverse mutational processes, such as exposure to ultraviolet light and the deamination of methylated cytosines, but largely also on the sequence content of different genomic regions on which these processes act. With MutViz (http://gmql.eu/mutviz/), we have presented a user-friendly web tool for the identification of mutation enrichments that offers preloaded mutations from public datasets for a variety of cancer types, well organized within an effective database architecture. Somatic mutation patterns can be visually and statistically analyzed within arbitrary sets of small, user-provided genomic regions, such as promoters or collections of transcription factor binding sites. Here, we present MutViz 2.0, a largely extended and consolidated version of the tool: we took into account the immediate (trinucleotide) sequence context of mutations, improved the representation of clinical annotation of tumor samples and devised a method for signature refitting on limited genomic regions to infer the contribution of individual mutational processes to the mutation patterns observed in these regions. We described both the features of MutViz 2.0, concentrating on the novelties, and the substantial re-engineering of the cloud-based architecture.

## Graphical Abstract

**Graphical Abstract:**
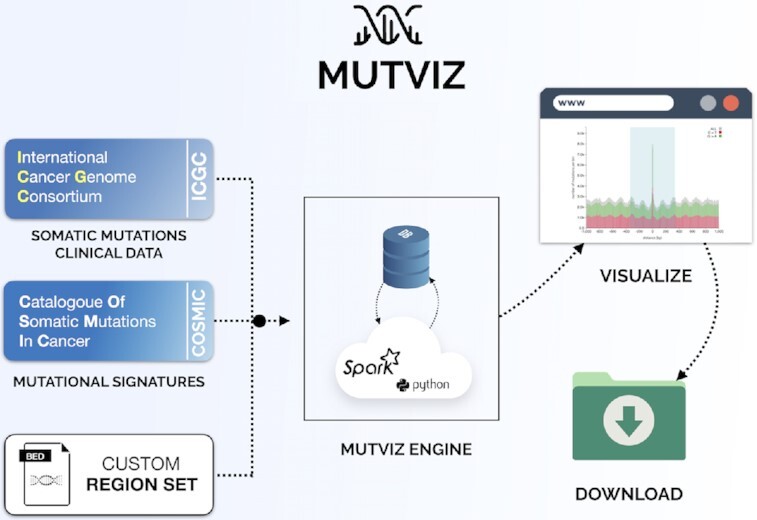
Simplified workflow of data analysis and visualization with MutViz: Analysis of somatic mutations within user-defined genomic regions for identification and visualization of region-specific mutation characteristics.

## INTRODUCTION

The relevance of somatic mutations in tumorigenesis is well established. While initial studies were mostly concentrated on protein-coding regions of the genome, the possibilities provided by next-generation sequencing techniques have spurred an increasing interest in non-coding regions. By now, the enrichment of mutations in many regulatory regions has been linked to cancer development, including mutations in estrogen receptor binding sites in breast cancer (1), recurrent microdeletions in CCCTC-binding factor (CTCF) binding sites involved in chromatin organization, which lead to a deregulation of oncogenes in T-cell acute lymphoblastic leukemia (2), and mutations in other transcription factor binding sites (3).

Recently, the development of frameworks to describe the activity of mutational processes (4–6)—including extrinsic carcinogens, such as ultraviolet light or tobacco smoke, and intrinsic processes, such as the spontaneous deamination of 5-methylcytosine—has provided an additional layer of mutation analysis for cancer genomics. The most commonly used framework describes the activity of mutational processes in terms of mutational signatures composed of 96 mutation probabilities of sequence triplets whose central base is altered (6 possible base changes × 4 possible adjacent bases at 5′ × 4 possible adjacent bases at 3′ = 96) (4,5,7).

**Figure 1.**
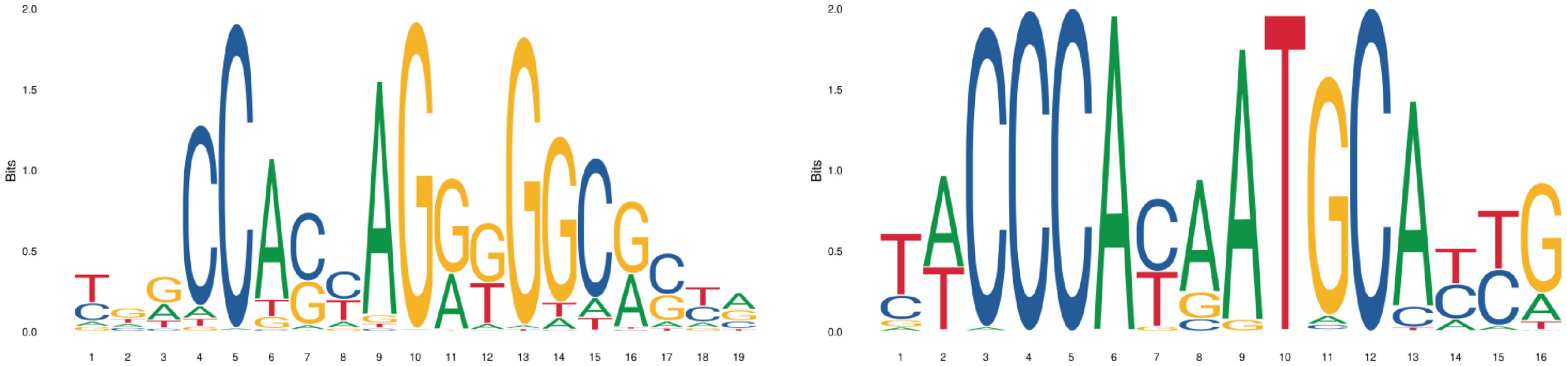
Sequence logos for the binding sites of CTCF (left) and ZNF143 (right). Obtained from Jaspar (13) with accession numbers MA0139.1 and MA0088.2.

In this context, it is important to understand that the various mutational processes can have largely different effects on different subsets of the genome—like coding regions, promoter regions, regulatory elements and so on—depending on both their functional constraints and their different sequence content (8–10). While, for instance, the cytosine to thymine (C→T) or cytosine–cytosine to thymine–thymine (CC→TT) changes associated with ultraviolet light (11,12) can mutate several positions in consensus CTCF binding sites (8) (see Figure 1; in particular positions 4–5, 7, 10, 13–15 and 17), the age-related spontaneous deamination of 5-methylcytosines (also C→T) actually requires the presence of CpGs (hence, CpG→TpG), which are usually much less abundant than cytosines in these binding sites (mostly at position 15/16; see Figure 1).

In our own previous work, we have investigated the enrichment of somatic mutations, abnormal methylation and copy number alterations in the proximity of active CTCF binding sites at the boundaries of topologically associated domains (TADs) in 26 cancer types (14), and developed a theoretical, mathematical framework to predict the impact of different mutational processes on binding sites of specific transcription factors (8).

These and other studies highlight the need of investigating the enrichment of somatic mutations—and the sequence context in which they occur—in arbitrary sets of genomic regions, rather than focusing only on mutations affecting the protein-coding portion of DNA, as often done in the past.

This motivated us to develop MutViz, a web tool for visual and statistical analysis of somatic mutations and their enrichment in user-provided sets of small genomic regions of selected cancer types (15). Comparative analysis regarding different types of single nucleotide variants, different sets of genomic regions and different cancer types is supported.

Since then, we have significantly improved MutViz’s architecture, database and functionality, including a new processing engine based on Apache Spark (16), new visualization options for more flexible inspection of mutational patterns in selected genomic regions and the possibility to perform signature refitting to determine the contribution of mutational signatures to the mutational patterns observed in these regions.

In this article, we first summarize the methods and the basic functionality of the tool, followed by a more detailed discussion of its new, extended functionality and its implementation. To show the tool’s utility by means of a use case, we analyze somatic mutations of CTCF binding sites in various cancer types.

## MATERIALS AND METHODS

### Mutation data

Somatic mutation data were obtained in January 2021 from the International Cancer Genome Consortium (ICGC; https://icgc.org/) and The Cancer Genome Atlas (TCGA; https://www.cancer.gov/tcga) through the ICGC Data Portal (https://dcc.icgc.org/). We collected more than 82 million somatic single nucleotide mutations from 53 different whole-genome sequencing tumor datasets (tumors of 6228 donors) and from 43 different whole-exome sequencing tumor datasets (tumors of 13 725 donors). A detailed list of datasets, cancer types and donor counts is provided in Supplementary Table S1.

### Mutational signatures

Mutational signatures (version 3.0, May 2019), as determined by Alexandrov *et al.* (7), were taken from the Catalogue of Somatic Mutations in Cancer (COSMIC) at https://cancer.sanger.ac.uk/cosmic/signatures. We integrated only single base substitution (SBS) signatures in MutViz 2.0, but excluded those that have been identified only in exome sequencing samples (SBS 23, 25 and 42) or characterized as possible sequencing artifacts (SBS 27, 29, 43 and 45–60).

### Transcription factor binding sites

To get users started, MutViz 2.0 provides a limited number of predefined sets of genomic regions, namely binding sites of several transcription factors. Each set of binding sites was identified starting from the position frequency matrix (PFM) of a specific transcription factor as provided by Jaspar 2018 (17). The sequence logos in Figure 1 are derived from such PFMs.

Using Biostrings (18) with a threshold of 80%, we identified potential binding sites matching the PFM in the human reference genome hg19. In order to focus on experimentally validated binding motifs, we subsequently used GMQL (19)—an in-house developed genomic query language—to intersect the predicted sites with ChIP-seq peaks for the H1-hESC, GM12878 or HeLa S3 cell line, downloaded from ENCODE (20).

### Statistical test for local mutation enrichment

**Figure 2.**
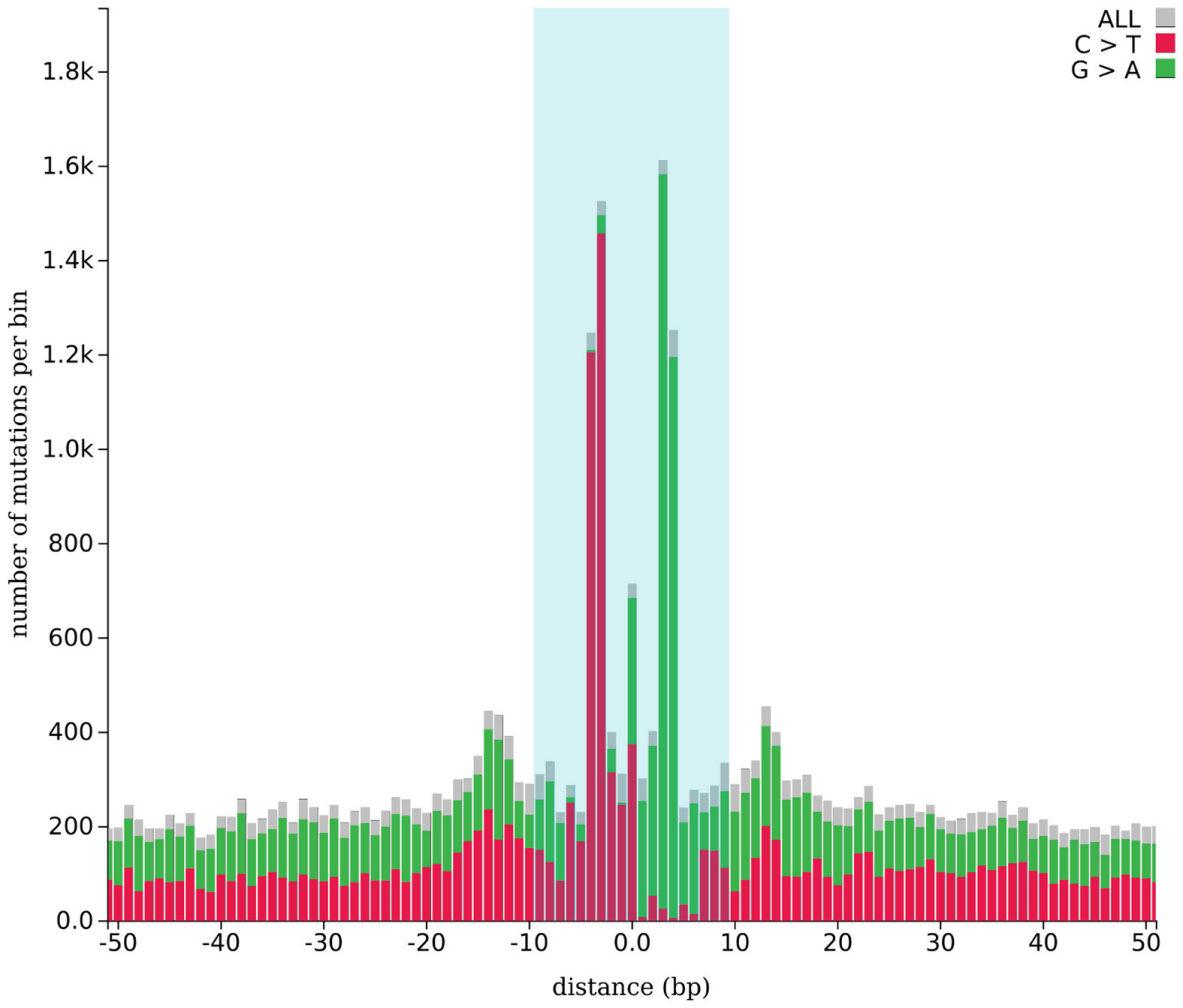
Histogram view with a bin size of 1. Somatic single nucleotide mutations located on or close to CTCF binding sites (indicated by a light blue background) in 183 melanomas. C→T base changes, and their reverse complement G→A mutations are highlighted. Positions on the *x*-axis refer to the forward strand; the actual binding sites (compare to Figure 1), however, may also lie on the reverse strand.

To test whether a specific set of regions (such as transcription factor binding sites) is affected by a significantly higher number of mutations (hypermutation) or a significantly lower number of mutations (hypomutation) than the corresponding flanking regions (see Figure 2), MutViz 2.0 performs a permutation test. For this purpose, all mutations falling within an adjustable window centered around regions from the input set—each window thus containing a region of interest plus its flanking regions—are randomly re-positioned across the window. This random distribution of mutations is repeated 10 000 times, and a *P*-value is determined according to the fraction of permutations which assign the regions of interest a number of mutations equal to or greater than the true mutation count (for hypermutation), or alternatively a number of mutations equal to or lower than the true mutation count (for hypomutation). By default, the window size is double the size of the largest region of interest with a minimum of ±1 kbp (for regions smaller than 1 kbp) and a maximum of ±5 kbp (for regions exceeding 5 kbp) around the regions’ central points. Both the window size for flanking sequences and for the central region in which to search for enrichment (usually the regions of interest, e.g. binding sites) can be adjusted by the user.

This approach tests for local enrichment only, directly comparing regions of interest with their flanking regions. We thereby avoid, or at least reduce, the potential bias to be expected when comparing genomic regions with totally different accessibility or epigenetic features.

### Statistical test for comparing region sets or tumor types

**Figure 3.**
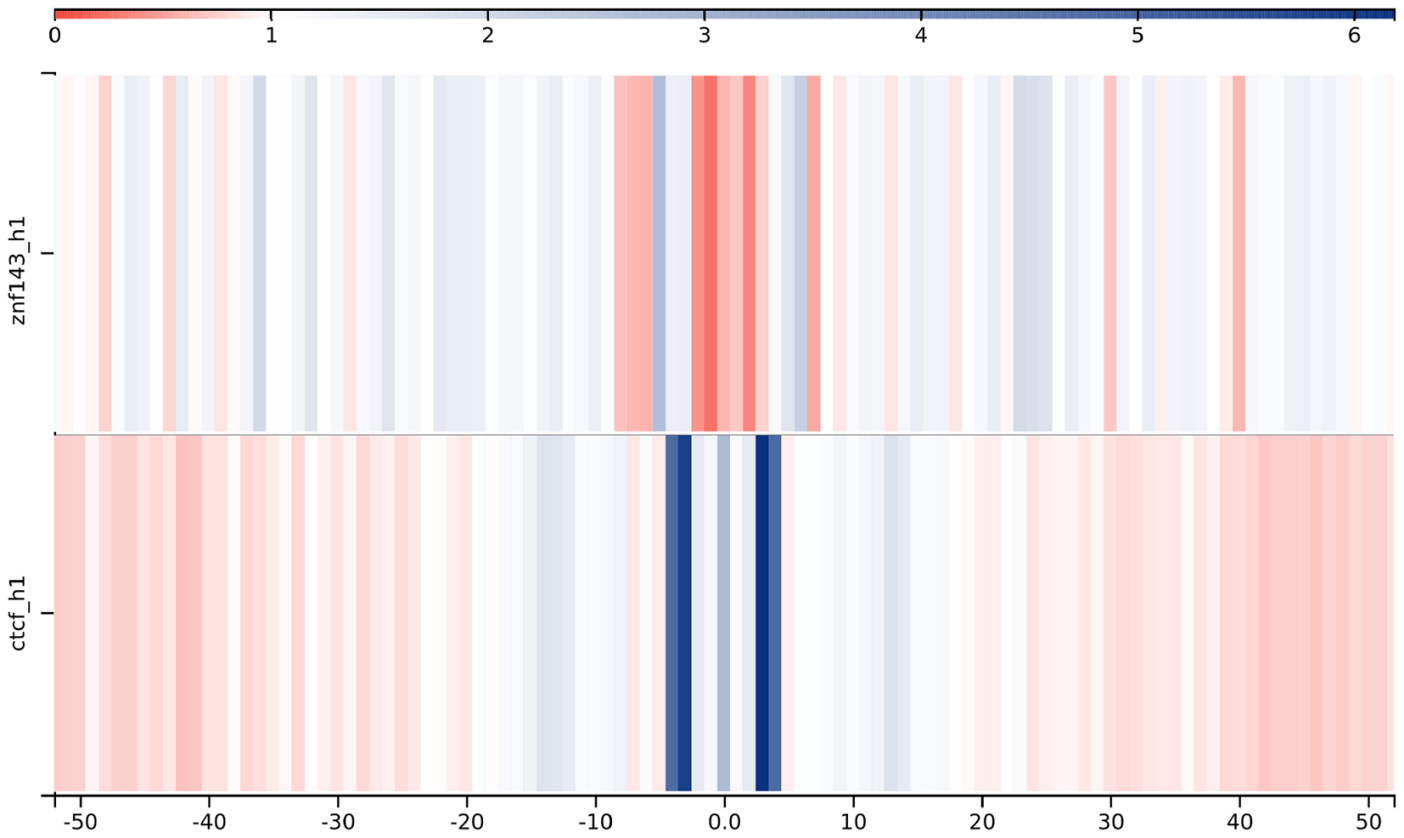
Region comparison view with a bin size of 1. Somatic single nucleotide mutations located on or close to binding sites of ZNF143 (top) and CTCF (bottom) in 183 melanomas. The color intensity scale, shown above the region sets, indicates fold-enrichments (observed/expected ratios) with respect to the average mutation rate in the targets plus flanking regions.

**Figure 4.**
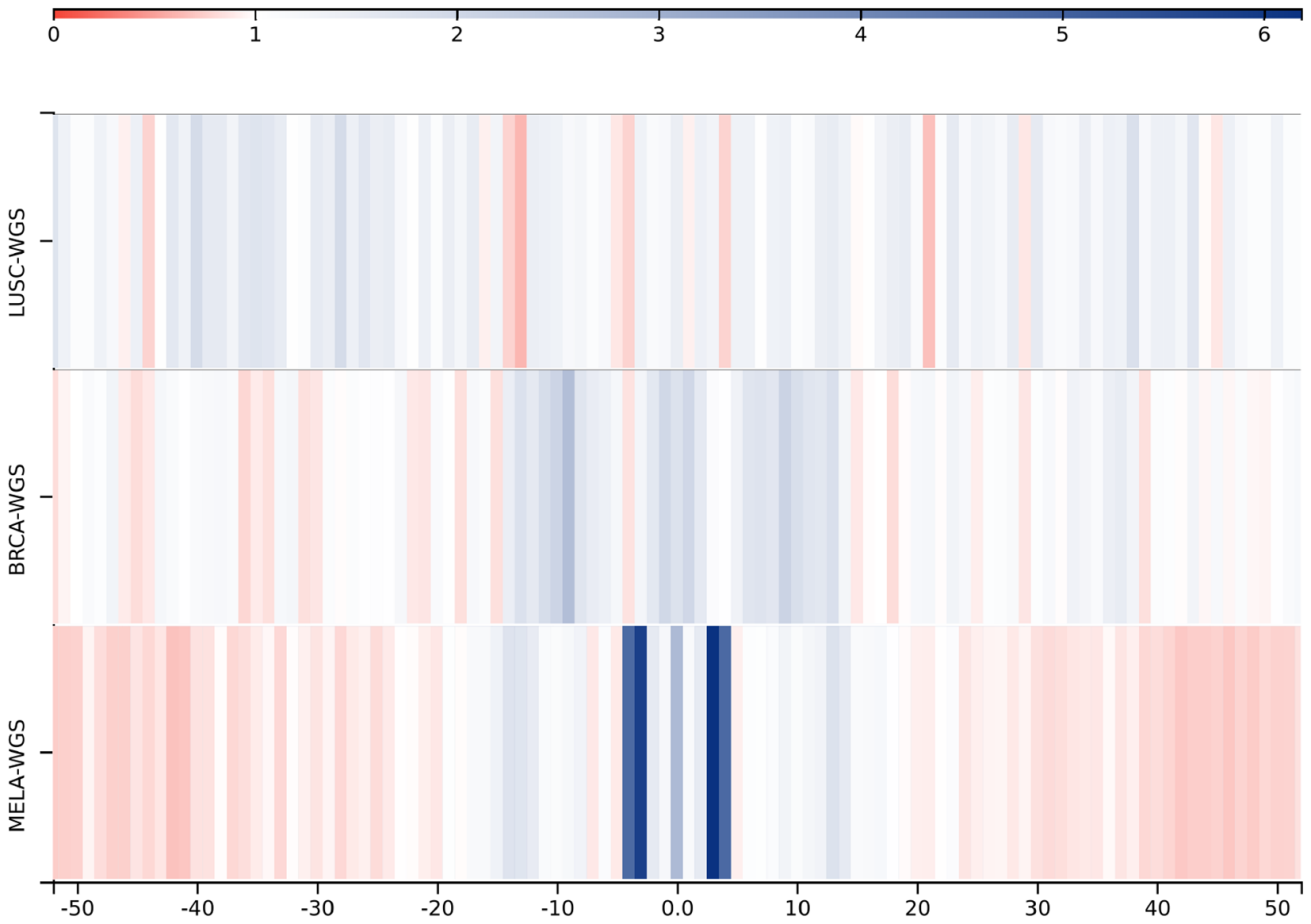
Tumor comparison view with a bin size of 1. Somatic single nucleotide mutations located on or close to CTCF binding sites in 78 lung squamous cell carcinomas (LUSC), 777 breast cancers (BRCA) and 183 melanomas (MELA). The color intensity scale, shown above the region sets, indicates fold-enrichments (observed/expected ratios) with respect to the average mutation rate in the targets plus flanking regions.

To identify statistically significant differences between the number of mutations falling within two different region sets in the same tumor type (see Figure 3), or of the same region set in two different tumor types (see Figure 4), we perform a χ^2^ test of independence of variables according to the contingency table *C* = [[*f*_1_, *r*_1_], [*f*_2_, *r*_2_]], where each row contains the average number of mutations per base in the flanking regions (*f*_*n*_) and in the regions of interest (*r*_*n*_) of the two conditions (region sets or tumor types) to be compared.

In other words, the numbers of mutations are not compared directly, but with respect to their respective flanking regions. This as well serves to reduce bias due to the expected differences in accessibility and epigenetic features between the two conditions.

### Signature refitting for limited genomic regions

Given a set of mutational signatures *S* and a catalog of somatic mutations *M* found in a given tumor genome, an exposure (or contribution) *e*_*k*_ is computed for each signature *s*_*k*_ ∈ *S*, such that the exposure-weighted sum of signatures reflects as closely as possible the distribution of mutation types 〈*m*_*j*_〉_*M*_ observed in *M*:

$$\begin{equation*} \langle m_j\rangle _M \approx \sum _k e_k \times s_k \qquad \mathrm{with} \,\, \sum _k{e_k}=1 \,\, \mathrm{and} \,\, e_k\ge 0 \end{equation*}$$

Here, *m*_*j*_ is the fraction of observed mutations which are of the specific mutation type *j*. For the signature model introduced by Alexandrov *et al.*, *j* is one of the 96 mutation types within their trinucleotide context.

This procedure is often referred to as ‘signature refitting’ (21) and usually performed using the maximum number of mutation observations available for the given tumor sample. Indeed, most of the mutational signatures provided by COSMIC were derived from whole-genome sequencing data and are thus normalized to the trinucleotide frequencies of the whole genome (21).

Since the trinucleotide frequencies in potentially small genomic regions of interest can differ greatly from the genome-wide frequencies (8), it is not appropriate to directly apply (genome-wide) mutational signatures to the limited set of mutations located within these regions.

We therefore devised a novel approach by adjusting the mutational signatures to the trinucleotide content of the specific input regions. We determine a set of correction factors to be applied to the catalog of mutational signatures before performing signature refitting.

For each possible trinucleotide mutation type *j* (e.g. A[C→T]G) and the corresponding trinucleotide *t*(*j*) (e.g. ACG) which is altered by a mutation of type *j*, we determine a correction factor as:

$$\begin{equation*} c_j = c_{t(j)} = \frac{ \Big ( \frac{\# \mathrm{occurrences\,of\,} t(j) \mathrm{\,in\,} R}{\# \mathrm{trinucleotides\,in\,} R} \Big ) }{ \Big ( \frac{\# \mathrm{occurrences\,of\,} t(j) \mathrm{\,in\,} G}{\# \mathrm{trinucleotides\,in\,} G} \Big ) } \end{equation*}$$

where *R* is the region set of interest and *G* is the whole genome.

We then can adjust each mutational signature *s*_*k*_ and its mutation probabilities *s*_*k*_(*j*) for the individual mutation types *j* as follows:

$$\begin{equation*} s^{\prime }_k(j) = c_j \times s_k(j) \end{equation*}$$

with subsequent normalization

$$\begin{equation*} s_k^{adj}(j) = \frac{s^{\prime }_k(j)}{\sum _j{s^{\prime }_k(j)}} \end{equation*}$$

such that $\sum _j{s_k^{adj}(j)}=1$ for the adjusted signature $s_k^{adj}$.

This means, if a trinucleotide is more frequent in the region set than in the genome as a whole, we accordingly increase the associated mutation probability of the signature. Indeed, the region set provides a higher mutation opportunity (22) for this trinucleotide, such that in these regions the mutational process is likely to produce more associated mutations than on average in the rest of the genome. Likewise, if the trinucleotide is less frequent, we lower the associated mutation probability.

We can then use the set of adjusted signatures $S^{adj} = \lbrace s_k^{adj}\rbrace _k$ to perform signature refitting as outlined above, applying the quadratic programming approach we have successfully used before (23), to identify a set of exposures *e*_*k*_ which minimize the error between 〈*m*_*j*_〉_*M*_ and $\sum _k e_k \times s_k^{adj}$.

## RESULTS AND DISCUSSION

MutViz 2.0 users can define custom sets *R* of genomic regions *r*_*i*_ ∈ *R* in their workspace by uploading the coordinates of the respective genomic loci in a BED file format, using only the mandatory fields, i.e. without information on directionality. To perform a mutational analysis, several mutation datasets of various tumor types, collected from public repositories (see ‘Materials and Methods’ section), can be chosen. A small list of predefined genomic regions, including experimentally validated binding sites for CTCF and zinc-finger protein 143 (ZNF143; see Figure 1), allows to test MutViz 2.0 without previous requirements.

Based on the provided or chosen data, MutViz 2.0 maps the somatic mutations of a set of tumors to the specified genomic regions and provides visualization features that ease the comparative analysis between different mutation types (e.g. alterations of cytosines to thymines, C→T and cytosines to guanines, C→G), different sets of genomic regions (e.g. binding sites of different transcription factors) and different tumor types.

The visualization modes of MutViz 2.0 can be divided into two categories, based on the underlying computation required to construct them: (i) *distance-based* visualizations and (ii) *intersection-based* (or cumulative) visualizations. The former, already present in the previously published version of the tool (15), align the regions of interest *r*_*i*_ ∈ *R* with respect to their central points (defined by $r_{i_{\text{center}}} = \lfloor (r_{i_{\text{start}}} + r_{i_{\text{stop}}})/2\rfloor$) and evaluate mutations according to their distances from the center. The latter are novel features of MutViz 2.0 and count the number of mutations which overlap with the user-provided regions of interest, irrespective of their precise positions within these regions.

With the new version, the results of all visualization modes can be downloaded either as image files or in JavaScript Object Notation (JSON; https://json.org/) format for further downstream processing.

To illustrate the functionality of MutViz 2.0, in the following we analyze the enrichment of somatic mutations located on (or close to) CTCF binding sites in whole-genome sequencing data of 183 melanomas, and compare them to those regarding other tumor types or ZNF143 binding sites.

### Distance-based visualization modes

#### Histogram view

The position-wise overall count of somatic single nucleotide variants falling onto or close to the regions of interest can be visualized as a histogram, and a statistical test for the local enrichment of mutations can be performed (see ‘Materials and Methods’ section). The user can specify whether the test should be restricted to a particular class of base changes, e.g. only C→* mutations (i.e. all cytosine mutations, irrespective of the precise variant nucleotide). Figure 2, for example, clearly shows that in melamona CTCF binding sites are significantly more often mutated than their flanking sequences (*P* < 10^−4^) and that the vast majority are C→T base changes (including the reverse complement G→A), with the two highest peaks corresponding to mutations (on the forward and the reverse strand) of positions 13 and 14 of the CTCF motif shown in Figure 1. This enrichment can partly be explained by the C→T and CC→TT mutations associated with ultraviolet light (8,24) and has been observed also in other skin cancers (25).

#### Region comparison view

The somatic mutations of a given mutation dataset that are located on or close to two different region sets, such as binding sites of different transcription factors, can be directly compared. This allows to verify, for example, whether an accumulation of mutations is present in only one of the two. The accumulation of mutations w.r.t. their distances from the center of the regions of interest is visualized in form of a heatmap as illustrated in Figure 3. The figure shows that the mutational patterns observed across CTCF binding sites in melamona differ from those observed across ZNF143 binding sites. Indeed, while several positions within and around the CTCF binding motif show a significant increase in mutation rates, for most positions in the ZNF143 binding motif we can actually observe a depletion of mutations (see the histogram views in Figure 2 and Supplementary Figure S3).

#### Tumor comparison view

Instead of comparing two different region sets within the same mutation dataset, users can compare somatic mutations associated with a single region set but observed in two or more different mutation datasets (e.g. different tumor types). Figure 4 shows a heatmap which evidences that the strong accumulation of mutations in CTCF binding sites that can be observed in melamona is not present in lung squamous cell carcinoma (*P* = 2.91 × 10^−3^ in a pairwise comparison). While for breast cancer, the slightly more intense color close to the center adumbrates a minimal increase of mutation rates, in comparison to melamona, this increase is negligible (*P* = 5.71 × 10^−3^ in a pairwise comparison).

### Intersection-based visualization modes

For MutViz 2.0, we have designed and implemented several new visualization features to analyze mutations which are located on (i.e. intersect with) the regions of interest. For these features, we introduced the possibility to filter tumor samples according to dataset-specific clinical annotation—extracted from the ICGC portal—such as donor age, therapy and survival time. The user can build simple predicates such as: (donor_sex = ’M’) AND (first_therapy_type IN {’chemotherapy’, ’surgery’}). Each time the predicate is modified, the tool instantaneously indicates how many donors match that predicate.

#### Trinucleotide mutation view

Given all somatic single nucleotide mutations falling within the genomic regions of interest in a chosen set of tumors, with this new visualization mode it is possible to easily obtain the aggregate mutation counts for the possible 96 trinucleotide mutation types, i.e. in terms of the six possible classes of base substitution (C→A, C→G, C→T, T→A, T→C, T→G; not considering redundancy due to the reverse complement) and their immediately adjacent bases. Depending on the affected trinucleotide, a base change from cytosine to adenine, for example, would therefore be assigned to one of the sixteen distinct classes: A[C→A]A, A[C→A]C, …, T[C→A]G, T[C→A]T.

**Figure 5.**
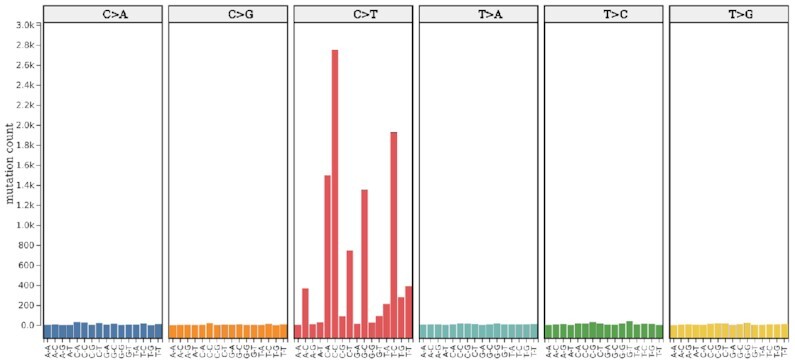
Trinucleotide mutation view. Total counts of somatic single nucleotide mutations (within their trinucleotide context) located on CTCF binding sites in 183 melamonas. The possible base changes are shown in six different sections (without redundancy due to reverse strand); the flanking bases are shown on the *x*-axis.

Figure 5, for example, shows that the vast majority of single nucleotide mutations of CTCF binding sites in melamona are cytosine-to-thymine mutations (C→T). At the same time, the low counts of A[C→T]G, C[C→T]G and G[C→T]G, and the only moderate count of T[C→T]G evidence that only a small fraction of these mutations fall on CpGs and can hence be caused by the spontaneous deamination of 5-methylcytosine already mentioned above. This is consistent with the hypothesis that the majority of somatic CTCF binding site mutations in melamona are caused by ultraviolet light (8).

Of note, the distribution of mutation types produced by this visualization mode is equivalent to the representation of mutational signatures developed by Alexandrov *et al.* (4,5,7) and provided by COSMIC (see ‘Materials and Methods’ section). It can thus be easily compared to the mutational patterns produced by various mutational processes (see also ‘Signature refitting view’ section below).

#### Mutations per donor view

**Figure 6.**
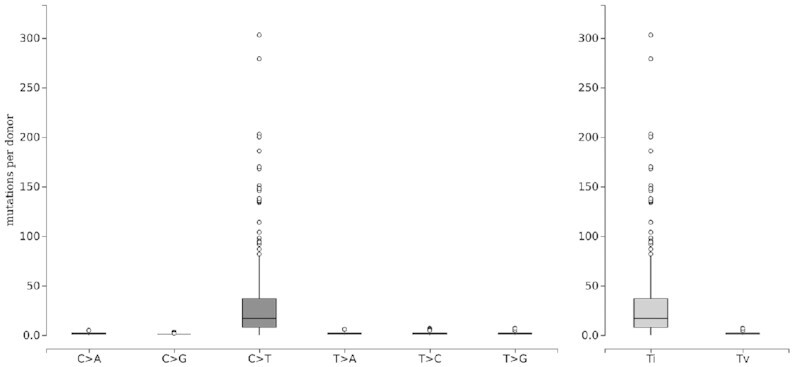
Mutations per donor view. Box plot of the number of somatic single nucleotide mutations located on CTCF binding sites in 183 individual donors with melamona. Right panel: number of transitions (Ti) versus transversions (Tv) in individual donors.

The trinucleotide mutation view aggregates somatic mutations over an entire set of tumors. In some cases, it may instead be of interest to get an understanding about how frequently different mutation types are observed in the individual tumors of a dataset. For this purpose, we implemented a further visualization mode which does not show a single summary bar for each mutation type (as in Figure 5) but instead a box plot which represents the distribution of the number of mutations observed in the different donors. This view can be produced for the six different base changes, as shown in Figure 6, that also reports the number of transitions (purine→purine or pyrimidine→pyrimidine) versus transversions (pyrimidine→purine or purine→pyrimidine). Alternatively, this mode can produce box plots for all 96 mutation types in their trinucleotide context (similar to Figure 5 but with box plots; not shown here).

Hovering the mouse pointer over the central box or over individual outliers of a box plot, the user can get further information such as the minimum, maximum and median number of mutations, or the donor ID and precise mutation count, respectively.

#### Signature refitting view

Different mutational processes can generate different mutation patterns. Even when they cause the same base changes, these need not necessarily occur in the same sequence context, such as the C→T mutations generated by ultraviolet light and the spontaneous deamination of methylated cytosines, as outlined above.

Given a set of well-defined mutational signatures and the mutation catalog observed in a specific tumor sample, it is possible to infer the most likely exposure of the tumor to the individual mutational signatures, and thus to the individual mutational processes. This procedure is often referred to as ‘signature refitting’ (21). The computed ‘exposures’ (or contributions) estimate what fractions of the observed mutation load of the tumor can be attributed to the different mutational signatures.

Since MutViz 2.0 applies signature refitting to a limited subset of mutations, namely those located within the user’s regions of interest, it adjusts mutational signatures according to the differences in trinucleotide frequencies between these regions and the whole genome (see ‘Materials and Methods’ section).

**Figure 7.**
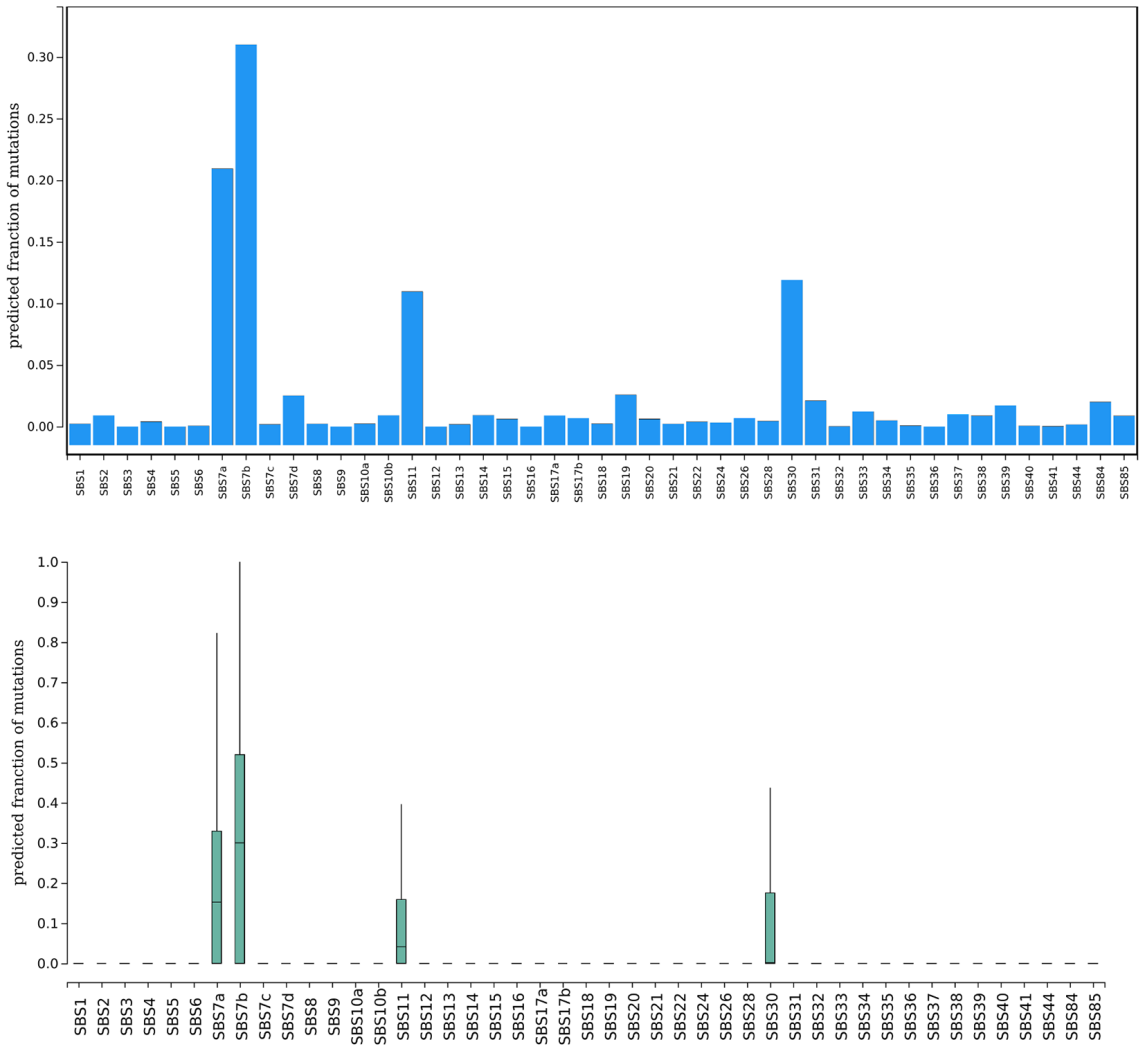
Signature refitting view. Predicted percent contribution of individual mutational signatures to the somatic single nucleotide mutations observed at CTCF binding sites in melamona. Upper plot: average percent contribution as a bar plot; lower plot: distribution of percent contributions in individual donors as a box plot.

Figure 7 shows the exposures obtained for CTCF binding sites in melamona, which estimate the likely contributions of individual mutational signatures to the somatic mutations observed in these binding sites. Indeed, the strongest SBS signatures are SBS7b and SBS7a (contributing on average over 30 and 20% of mutations, respectively), which are known to be associated with ultraviolet light (7) and were predicted by our theoretical framework to be the major contributors (8).

Although this result was achieved with only a very limited subset of mutations, its accentuation of mutational processes caused by ultraviolet light is congruent with the result obtained for the full set of mutations from the whole genome, which we presented in our previous work for the same tumor set (8). Considering the whole genome, however, signature SBS7a (about 51%) is stronger than SBS7b (about 15%). A confounding factor, that may explain the better scoring of SBS7b when looking at CTCF binding sites alone, is that their highly abundant trinucleotides CCA, CCC and CCT are more likely altered by SBS7b, while the trinucleotides mostly affected by SBS7a (TCA, TCC and TCG) are less frequent in CTCF binding sites (8). Therefore, using only a very limited subset of mutations with region-adjusted mutational signatures is ultimately only an approximation and may lead to slight inconsistencies.

Also signature SBS11, which is likely related to treatment with the alkylating agent temozolomide and appears to contribute on average about 11% of the mutations found in CTCF binding sites, has previously been identified in a subset of malignant melamonas (4,7). Signature SBS30, however, which shows an average of about 12% in Figure 7 and has been associated with deficiency in base excision repair due to inactivating mutations in NTHL1, has not been observed in melamonas.

How well region-specific signature refitting—i.e. refitting based only on a region-specific subset of mutations—can approximate the exposures obtained for whole-genome signature refitting (using mutation information from the entire tumor genome) depends on the number of region-specific mutations available for signature refitting. But even for low mutation counts, as we demonstrate in the Supplementary Material, adjusting signatures to the trinucleotide content of the regions of interest prior to refitting can significantly improve the results.

### CTCF binding site mutations at TAD boundaries

An important finding for several cancer types, which can be explored with the help of MutViz 2.0, is a significant enrichment of somatic mutations in active CTCF binding sites that fall onto boundaries of TADs—thus being involved in CTCF-mediated insulation of neighboring domains—as opposed to CTCF binding sites that are active but located at other genomic loci (14).

We took information on the activity of CTCF binding sites from ChIP-seq data for the H1 human embryonic stem cell line (H1-hESC) and separated active sites into those that are located at TAD boundaries (‘in-boundary’) according to ChIA-PET (chromatin interaction analysis by paired-end tag sequencing) experiments (26) and those that are not located at TAD boundaries (‘off-boundary’). For further details, please see the Supplementary Material.

**Figure 8.**
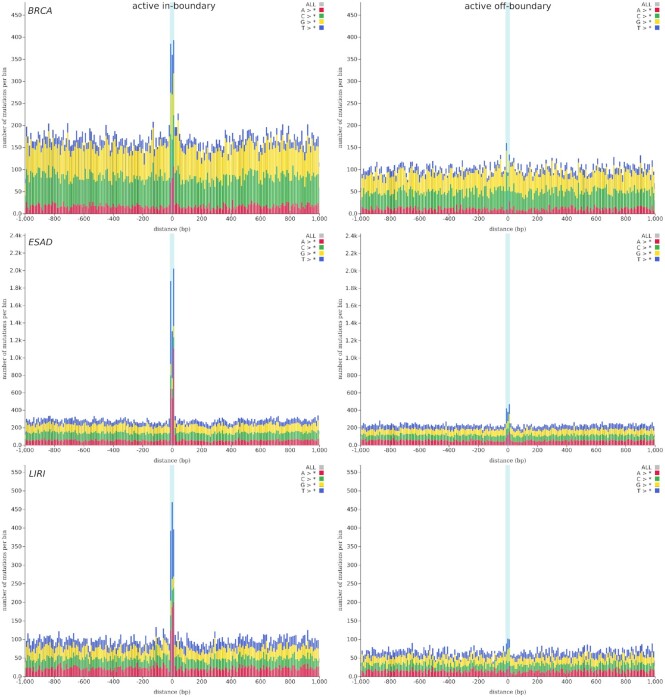
Somatic mutation enrichment at active CTCF binding sites (highlighted by a light blue background) as compared to their flanking genomic regions (−1 to +1 kb from the center of each binding site) at TAD boundaries (‘in-boundary’; left) and other genomic locations (‘off-boundary’; right). Results are shown for three whole-genome sequencing tumor datasets: breast cancer (BRCA), esophageal adenocarcinoma (ESAD) and liver cancer (LIRI).

The results, illustrated in Figure 8 for three tumor types, evidence that observed mutational rates of transcription factor binding sites can strongly depend on the cellular function they are involved in. Indeed, while active off-boundary binding sites for CTCF show at best a marginal increase in mutation rates with respect to flanking genomic regions, active in-boundary sites are clearly much more frequently mutated in breast cancer, esophageal adenocarcinoma and liver cancer. This suggests that the disruption of insulated neighborhoods may play an important role in these tumors (14).

### Implementation

The design of MutViz 2.0 was guided by two important requirements: first, the need to efficiently process a large quantity of somatic mutations and compute summary statistics and statistical tests for potentially large sets of uploaded genomic regions of interest; and second, the desire to provide an easy-to-use web application, backed by a responsive and intuitive user interface with advanced visualization features.

**Figure 9.**
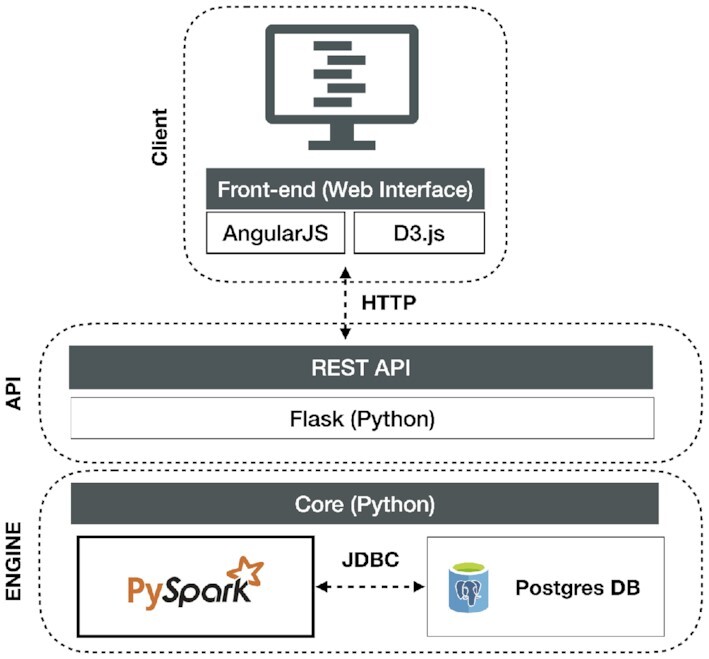
The three-tier architecture of MutViz 2.0.

Thus, we developed a three-tier architecture (see Figure 9) composed of

- a PostgreSQL-based data layer that stores both mutations and (temporarily) user-provided region sets, as well as cached results that avoid repeated computations on the same inputs;
- a core application layer (backend), implemented in Python and accessible via a REST API, that manages database queries and the functional logic, initiates parallel data processing via Apache Spark (16) and controls the information flow between all layers; and
- a front-end presentation layer that allows users to interact with the tool and visualize the results in a standard web browser.

The realization of the new intersection-based visualization modes has required a substantial re-engineering of the MutViz architecture, including the extension of the mutation repository to include information about the flanking base pairs. It also motivated the implementation of a new subsystem that partitions the mutation dataset for parallelizing the identification of mutation–region intersections, including a parallel and scalable algorithm specifically designed for this purpose. Further details about the software architecture, the used database schema and the processing of user queries are described in the Supplementary Material.

While the MutViz2 web application hosted at our website (http://gmql.eu/mutviz/) can be used only for custom region sets—relying on pre-loaded and pre-processed mutation data from ICGC and TCGA—the source code of the tool is freely available and allows for local installations based on own mutation databases.

## CONCLUSION

In this article, we have presented a significantly improved and extended version of MutViz, a web application that can support cancer research by analyzing regions of interests, such as transcription factor binding sites, for enrichment in single nucleotide mutations. The new features add the opportunity to consider these mutations within their immediate sequence context, and provide a novel approach to perform approximate signature refitting using only the limited number of mutations located within the selected regions of interest.

## DATA AVAILABILITY

MutViz 2.0 is publicly available as a readily usable web application at http://gmql.eu/mutviz/ and as an open-source project at https://github.com/DEIB-GECO/MutViz.

## Supplementary Material

zcab012_Supplemental_File
