# Supplementary material for: MutViz 2.0: visual analysis of somatic mutations and the impact of mutational signatures on selected genomic regions
Source: NAR Cancer. 2021 Apr 9;3(2):zcab012. doi: 10.1093/narcan/zcab012 (PMC8210215; doi:10.1093/narcan/zcab012)
Supplement: zcab012_Supplemental_File [file zcab012_supplemental_file.pdf]

Supplementary material for “MutViz 2.0: visual analysis of somatic mutations and the impact of mutational signatures on selected genomic regions”

Andrea Gulino [1,*], Eirini Stamoulakatou [1]
and Rosario M. Piro [1,*]

[1]Dipartimento di Elettronica, Informazione e Bioingegneria (DEIB), Politecnico di Milano, Via Ponzio 34/5, 20133 Milan, Italy

*To whom correspondence should be addressed:
Email: {andrea.gulino, rosariomichael.piro}@polimi.it

February 2021

Contents

1 Software architecture **3**

2 Database schema **5**

3 Processing of user queries **7**
3.1 Queries for distance-based visualizations 7
3.2 Queries for intersection-based visualizations 7

4 User Interface **10**

5 Mutation data and region sets **11**

6 Supporting plots and analyses **13**
6.1 ZNF143 binding site mutations in melanoma 13
6.2 Signature refitting for limited subsets of the genome 13
6.2.1 Regions sets and mutations 13
6.2.2 Signature refitting 14
6.2.3 Explained variance of exposure vectors 15
6.2.4 Accuracy of exposure vectors 15

7 Example study: CTCF binding site mutations at TAD boundaries **21**
7.1 Introduction: Topologically associated domains and cancer . . . 21

7.1.1 Result: enrichment of CTCF binding site mutations at TAD boundaries 21
7.2 Result: types of mutations in CTCF binding sites at TAD boundaries in esophageal adenocarcinoma 23

List of Figures

1 Three-tier architecture of MutViz 2.0 3
2 Logical schema of the database 5
3 Histogram view of mutations in ZNF143 binding sites in melanoma 13
4 Distribution of the number of mutations in promoters and CTCF binding sites in melanoma 14
5 Explained variance with and without signature adjustment . . . 16
6 Distribution of cosine similarities with and without signature adjustment 17
7 Cosine similarities for individual donors with and without signature adjustment 18
8 Distribution of MSE with and without signature adjustment . . . 19
9 MSE for individual donors with and without signature adjustment 20
10 Somatic mutations of active CTCF binding sites and TAD boundaries 22
11 Trinucleotide mutation distribution for active, in-boundary CTCF binding sites in ESAD 23
12 Number of specific base changes per patient in CTCF binding sites 24

List of Tables

1 Somatic mutation datasets from ICGC and TCGA 11
2 Jaspar 2018 matrix profiles for pre-loded regions sets 12

1 Software architecture

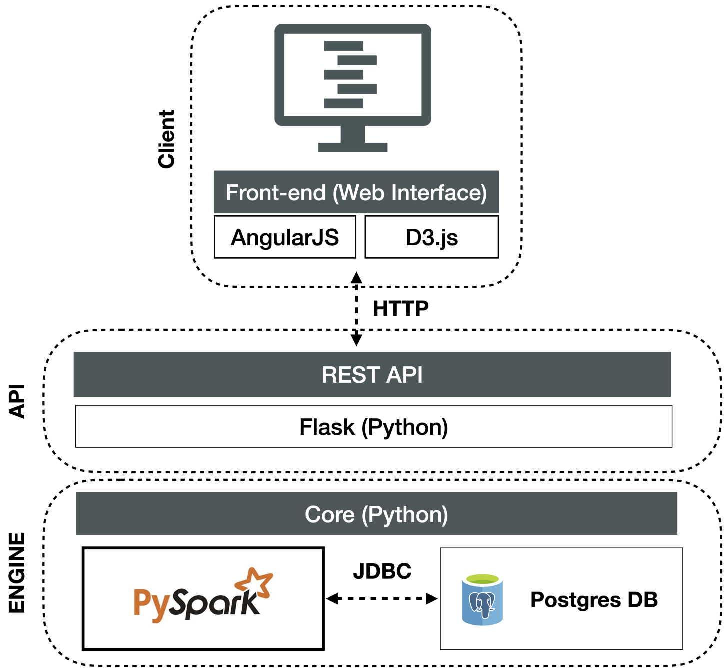

Supplementary Figure 1: The three-tier architecture of MutViz 2.0.

MutViz 2.0 is organized in a three-tier architecture (see Supplementary Fig. 1):

- **Front-end** (presentation layer): provides all the interactive visualizations described in our paper, implemented using the Angular.js[1] framework and D3.js.[2]
- **Middle Tier** (backend, core layer): made of a REST API backed by the MutViz 2.0 engine. The engine, implemented in Python, performs the main computations, i.e., mutation–region intersections, mutation–region distances and signature refitting. The engine makes results available through a simple REST API. Depending on the type of computation and on the availability of cached results, the engine will choose whether to run an SQL query on the database (using SQLAlchemy[3]) or start a parallel algorithm implemented on PySpark[4]. The REST API, through which the core layer can be accessed by the front-end, is implemented using the Flask[5] micro-framework.
- **Storage** (data layer): made of a Postgres database and a file repository. The database includes the mutation table, user region files (which are

[1]https://angularjs.org
[2]https://d3js.org
[3]`https://www.sqlalchemy.org/`
[4]https://spark.apache.org/
[5]https://flask.palletsprojects.com

stored only temporarily), pre-loaded region files, clinical data, and cached results that avoid repeated computations on the same inputs. The mutation table is also stored as a file on the Hadoop Distributed Filesystem[6] for processing with PySpark. For more details on the database, see Section 2.

[6] https://hadoop.apache.org/

2 Database schema

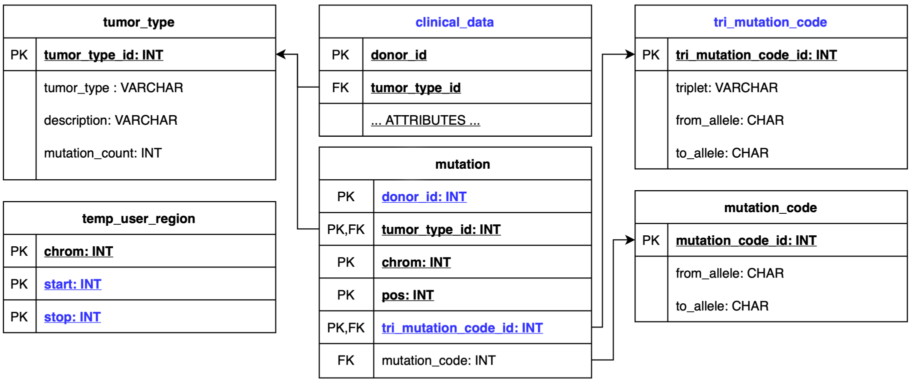

Supplementary Figure 2: Logical schema of the MutViz 2.0 database. Somatic single nucleotide mutations are stored in a persistent main table (here represented by `mutation`) with auxiliary tables, while user-provided regions are inserted in the temporary table `temp_user_region` and dropped at the end of the session. "...ATTRIBUTES..." in the table `clinical_data` stands for a longer list of clinical annotations such as gender, age and therapy. Tables and items highlighted in blue have been added for the new features of MutViz 2.0 and were not present (or implemented differently) in the previous version.

The MutViz 2.0 data layer organizes and stores the somatic mutation data, relying on the open-source relational database management system PostgreSQL. Supplementary Fig. 2 represents the logical schema of the database.

The main table, in Supplementary Fig. 2 represented with the name `mutation`, does actually have two implementations used for the two different visualization modes, `trinucleotide_mutation` for intersection-based visualizations and `mutation_ group` for distance-based visualizations:

- The table `trinucleotide_mutation` stores mutation data as individual mutations associated to specific donors and tumor types (or better: tumor datasets; not always different tumor types, see Section 5, Supplementary Table 1). For each specific somatic single nucleotide mutation, it associates the donor (patient) identifier, the tumor type/dataset (e.g., BRCA / breast cancer), the chromosomal position, and the associated base change within its trinucleotide sequence context (or "trinucleotide mutation code"). This allows to compute statistics regarding the number of trinucleotide mutations per donor and perform signature refitting on individual tumors.

- The second implementation of `mutation`, named `mutation_group`, groups mutations by tumor type, and does hence not require the data item `donor_id`. Instead, an additional data item `mutation_count` indicates how many donors have the specific base change at the given chromosomal position. Also, the trinucleotide context is not taken into consideration and the auxiliary table `tri_mutation_code` is replaced by the simpler base change representation in `mutation_code`. This aggregation of mutations

by tumor dataset speeds up the computation for distance-based visualizations where only total counts are needed and donor-specific details play no role.

In both `mutation_group` and `trinucleotide_mutation`, the tumor type and the base change (mutation code or trinucleotide mutation code, respectively) are encoded as integers. This reduces the size of each row, thereby decreasing the total number of page reads. The decoding of the mutation codes (base changes) and tumor type codes can be performed by a lookup in the satellite tables `mutation_code`, `tri_mutation_code` and `tumor_type`.

Clinical features and annotations of individual tumors are contained in the table `clinical_data` and matched to the donor/patient identifier and the tumor type/dataset.

Finally, the table `temp_user_region` is a temporary table, whose scope and persistence is limited to the single user session. For each region of interest, the table stores the chromosome and the start and stop position of the region.

3 Processing of user queries

We exploit indexing features and query optimization of PostgreSQL in order to quickly compute the result of user queries. Thanks to our data representation and choice of indexes, typical queries matching input regions with somatic mutations in a window of ± 1 kbp around the region center run in a short time interval of a few seconds up to one or two minutes (and only a few milliseconds when previous results have been cached), which is excellent considering the size of the mutation datasets. Queries for larger windows of up to ± 5 kbp can take up to several minutes. Still, this makes MutViz 2.0 a mostly fluid and responsive tool from the point of view of the user.

3.1 Queries for distance-based visualizations

First, the region set files provided by the user are loaded into the database as a temporary table, then the MutViz 2.0 backend/core layer opens a transaction through the database in order to run the following SQL query:

```
SELECT tumor_type_id,mg.pos - (t.start+t.stop)/2,
       mutation_code_id,sum(mutation_count)
FROM mutation_group AS mg
JOIN temp_user_region AS t
  ON mg.chrom = t.chrom AND
  mg.pos BETWEEN (t.start+t.stop)/2-@MAX AND
                 (t.start+t.stop)/2+@MAX
GROUP BY tumor_type_id,
         pos_dif, mutation_code_id
```

where `@MAX` represents the user-defined window size (as up- and downstream distance from the center point of the regions of interest).

This effectively computes the distances between the central point and all mutations within the window by simply executing an SQL query on the database.

3.2 Queries for intersection-based visualizations

For the new visualization modes, which are based on mutation–region intersections, we implemented an ad-hoc parallel processing algorithm based on PySpark, the Python API of Apache Spark.

It can take the user-provided region set and mutation data from the database described above (data are streamed from the database to Spark) or from CSV files stored on the filesystem.

The main steps of the procedure are:

1. The mutation dataset is split into an arbitrary, configurable number of partitions. Mutations from different chromosomes can belong to the same partition.

2. Within each partition, mutations are sorted by chromosomal position. Partitions can be sorted in parallel, independently of one another.

3. The set of user-provided regions of interest is sorted by chromosomal *start* position. Regions having the same start position are additionally sorted by their chromosomal *stop* position.

4. Each mutation partition is compared with a copy of the sorted regions of interest. Mutations which do not intersect with one of these user-provided regions are filtered out. Partitions can be matched to the regions of interest in parallel, independently of each other.

5. Finally, the results from the individual mutation partitions (mutations matching to the region set) are aggregated for the specific visualization mode (e.g., by donor for the “mutations per donor” view).

Algorithm 1: Mutation-region intersection algorithm

```
Algorithm intersection()
    Inputs: sorted partition the mutation-set M = {m_1, ..., m_|M|} and
     sorted region-set R = {r_1, ..., r_|R|}.
    Result: Mutations intersecting user-provided regions.
    idx_r = 0, idx_m = 0, result = ∅
    while idx_r < |R| and idx_m < |M| do
        if M[idx_m].pos < R[idx_r].start then
            // current mutation precedes current region
            idx_r = idx_r + 1
        else if M[idx_m].pos ≤ R[idx_r].stop then
            // current mutation overlaps the current region
            if M[idx_m].chrom == R[idx_r].chrom then
                result = result + M[idx_m]
            else
                result = result + lookAhead(M, R, idx_r, idx_m)
            end
            idx_m = idx_m + 1
        else
            // current region precedes the current mutation
            idx_r = idx_r + 1
        end
    end
    return result
Procedure lookAhead(M, R, idx_r, idx_m)
    result = ∅
    idx_next = idx_r + 1
    while idx_next < |R| ∧ M[idx_m].pos ≥ R[idx_next].start do
        if R[idx_next].chrom == M[idx_m].chrom ∧
         R[idx_next].stop ≥ M[idx_m].pos then
            result = result + M[idx_m]
        idx_next = idx_next + 1
    end
    return result
```

The intersection procedure is implemented in algorithm 1 and is based on classical line-sweep algorithms, adapted to support chromosome matching. Compared to standard algorithms, when a mutation and a region overlap but their chromosomes do not match, the mutation cannot be simply discarded,

since it could still match one of the upcoming regions. At the same time, the current region could overlap with the next mutations and cannot be discarded either. This problem is solved by the *lookAhead* step (lines 14–20, called in line 9) that performs a forward search, looking for regions that could match the current mutation.

This algorithm is efficient under the assumption that the number of regions in the user-provided region set is much smaller than the number of mutations in the database. This is the case even for frequently found transcription factor binding sites.

Filtering of mutation data according to clinical annotation (such as gender, age, therapy, etc.) is done a posteriori by joining the (cached) intersection result with the clinical data table from the database. Filtering the mutation data before computing the intersection instead would be less efficient because the resulting mutation set would be different for every user-defined filter on clinical annotation, and could therefore not be cached for following user queries with modified annotation filters.

4 User Interface

The user interface is an HTML 5 single-page application based on Angular JS[7] and D3.js[8]. It communicates with the application layer through the aforementioned REST API, retrieving processed data for building visualizations and computing statistical tests.

The interface is organized in eight main sections, namely:

1. **MutViz 2.0** (home): provides a description of the tool and the data on which it is built.

2. **Workspace**: allows users to manage their *workspace*, containing either uploaded region sets or example region sets from our public repository. The browser's local storage is used to save and recover the users' workspace.

3. **Histogram**: shows the distribution of mutations around the regions in a single region set.

4. **Region comparison**: provides a heatmap for comparing two different region sets for a given mutation dataset.

5. **Tumor comparison**: provides a heatmap for comparing two or more mutation datasets (e.g., different tumor types) for a given region set. A statistical test can be computed only when comparing exactly two mutation datasets.

6. **Trinucleotide mutations**: produces a bar plot showing the aggregated mutation counts for all 96 possible base changes within their trinucleotide sequence context (e.g., A[C→T]G); for one region set and one mutation dataset.

7. **Mutations per donor**: produces one of the following two alternatives:

   - a box plot showing the distribution of mutation counts within individual tumors for all 96 possible base changes within their trinucleotide sequence context (e.g., A[C→T]G); or
   - a box plots showing the distribution of mutation counts within individual tumors for the six basic classes of single nucleotide mutations, and additionally a box plot comparing the number of transitions (purine→purine or pyrimidine→pyrimidine) to the number of transversions (pyrimidine→purine or purine→pyrimidine).

8. **Signature refitting**: computes and visualizes exposures/contributions of mutational processes/signatures to the mutations observed in a given region set and mutation dataset. The visualization of exposures can either be a bar plot (average exposure across the donors for each mutational signature) or a box plot (distribution of exposures in individual donors for each mutational signature).

[7]https://angularjs.org
[8]https://d3js.org

5 Mutation data and region sets

Supplementary Table 1 reports the number of individual donors for each of the available mutation datasets.

Dataset	Tumor type	WGS donors	WXS donors
ALL	acute lymphoblastic leukemia	131	24
AML	acute myeloid leukemia	143	30
BLCA	bladder cancer	23	514
BOCA	bone cancer	162	65
BRCA	breast cancer	777	1182
BTCA	biliary tract cancer	71	239
CESC	cervical squamous cell carcinoma	20	289
CLLE	chronic lymphocytic leukemia	151	396
CMDI	chronic myeloid disorders	30	105
COAD	colon adenocarcinoma	44	397
COCA	colorectal cancer	30	291
DLBC	lymphoid neoplasm diffuse large B-cell lymphoma	7	37
EOPC	early onset prostate cancer	202	–
ESAD	esophageal adenocarcinoma	409	–
ESCA	esophageal cancer	17	315
GACA	gastric cancer	37	675
GBM	glioblastoma multiforme	41	382
HNSC	head and neck squamous cell carcinoma	44	507
KICH	kidney chromophobe	45	66
KIRC	kidney renal clear cell carcinoma	37	350
KIRP	kidney renal papillary cell carcinoma	33	272
LAML	leukemia (acute myeloid leukaemia and chronic myelogenous leukaemia)	32	391
LGG	brain lower grade glioma	18	508
LIAD	benign liver tumour	5	30
LICA	liver cancer	161	522
LIHC	liver hepatocellular carcinoma	54	362
LINC	liver cancer	31	363
LIRI	liver cancer	258	–
LMS	leiomyosarcoma	67	–
LUAD	lung adenocarcinoma	38	515
LUSC	lung squamous cell carcinoma	78	625
MALY	malignant lymphoma	241	–
MELA	melanoma	183	–
NACA	nasopharyngeal cancer	–	21
NBL	neuroblastoma	26	36
NKTL	T-cell and NK-cell lymphoma	30	–
ORCA	oral cancer	25	153
OV	ovarian cancer	135	411
PAAD	pancreatic cancer (adenocarcinoma)	–	177
PACA	pancreatic cancer (ductal adenocarcinoma)	454	315
PAEN	pancreatic cancer (endocrine neoplasms)	87	1
PBCA	pediatric brain cancer	638	42
PEME	pediatric medulloblastoma	112	–
PRAD	prostate adenocarcinoma	555	497
READ	rectum adenocarcinoma	16	140
RECA	renal cancer	95	–
RT	rhabdoid tumors	34	–
SARC	sarcoma (liposarcoma and multiple subtypes)	34	236
SKCA	skin adenocarcinoma	100	–
SKCM	skin cutaneous melanoma	37	466
STAD	gastric adenocarcinoma	38	439
THCA	thyroid cancer	48	783
UCEC	uterine corpus endometrial carcinoma	51	530
UTCA	uterine cancer (carcinosarcoma)	20	–
WT	Wilms tumor	73	26
Total:		6228	13725

Supplementary Table 1: Somatic mutation datasets from ICGC and TCGA. Donors without single nucleotide substitutions were excluded. WGS = whole genome sequencing; WXS = whole exome sequencing.

Transcription factor	Jaspar matrix profile
CTCF	MA0139.1
ELF1	MA0473.2
ELK4	MA0076.2
ETS1	MA0098.3
GABPA	MA0062.1
STAT3	MA0144.1
ZNF143	MA0088.2

Supplementary Table 2: Jaspar 2018 matrix profiles used for the pre-loaded region sets of transcritption factor binding sites.

6 Supporting plots and analyses

6.1 ZNF143 binding site mutations in melanoma

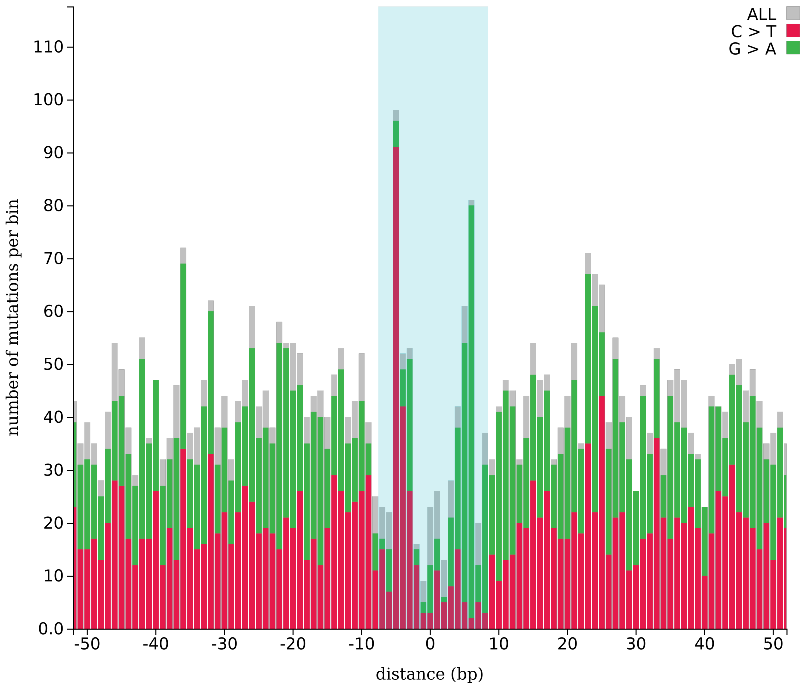

Supplementary Figure 3: Histogram view with a bin size of 1. Somatic single nucleotide mutations located on or close to ZNF143 binding sites (indicated by a light blue background) in 183 melanomas. C→T base changes, and their reverse complement G→A mutations are highlighted. Positions on the x-axis refer to the forward strand; the actual binding sites (compare to Fig. 1 in the paper), however, may also lie on the reverse strand. The two peaks of mutations within ZNF143 binding sites correspond to C→T changes—and the corresponding G→A changes on the reverse strand—at positions 3 to 5 of the binding motif (see the sequence logo in Fig. 1 of the paper).

6.2 Signature refitting for limited subsets of the genome

6.2.1 Regions sets and mutations

To evaluate whether signature refitting using only mutations from a limited subset of the genome (regions of interest) can be improved by adjusting mutational signatures according to the nucleotide content (i.e., the nucleotide frequencies) within the region set, we performed additional analyses for CTCF binding sites and promoter regions. The latter was chosen as an example for regions which are still limited in size but orders of magnitude larger than transcription factor binding sites, thus usually containing larger amounts of somatic mutations

as shown in Supplementary Fig. 4 for the melanoma (MELA) whole genome sequencing dataset.

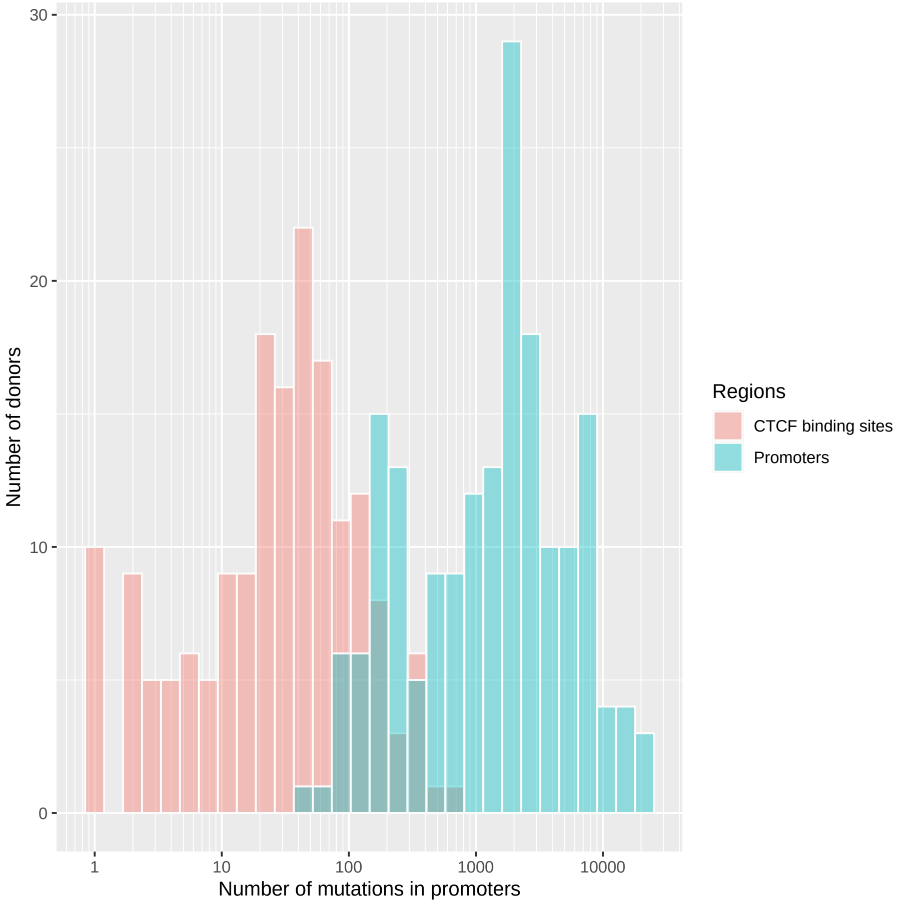

Supplementary Figure 4: Distribution of the number of somatic mutations falling within limited subsets of the genome in melanomas. Only donors with at east one mutation in the subset are shown (183 donors for promoters, 173 donors for CTCF binding sites).

We identified experimentally validated CTCF binding sites as described in the paper. As promoter regions we took all regions spanning from 2000 bases upstream to 200 bases downstream of transcription start sites (extracted from the R Bioconductor package `TxDb.Hsapiens.UCSC.hg19.knownGene`).

6.2.2 Signature refitting

We used the R/Bioconductor package `decompTumor2Sig` [1] and performed signature refitting (tumor genome "decomposition") for each individual donor using three different approaches:

1. **Whole-genome refitting**: using all somatic mutations from the entire genome with standard, unaltered mutational signatures (COSMIC v3.0 [2];

see our article). Since the obtained exposures are derived from the maximum information (i.e., maximum number of mutations) available for a donor, we took them as a baseline or “goal” for comparison with signature refitting based on limited subsets of regions (and hence subsets of mutations).

2. **Region-specific refitting without adjustment**: using only somatic mutations from a limited subset of regions (here: promoters or CTCF binding sites) *without prior adjustment* of mutational signatures according to the trinucleotide frequencies in the regions of interest.

3. **Region-specific refitting with adjustment**: using only somatic mutations from a limited subset of regions (here: promoters or CTCF binding sites) *with prior adjustment* of mutational signatures according to the trinucleotide frequencies in the regions of interest (as described in the Section “Signature refitting for limited genomic regions” of our article).

Each of these signature refitting procedures returns an exposure vector containing one exposure (i.e., percent contribution) for each mutational signature. The exposure vectors obtained from region-specific refitting can be compared with those obtained for whole-genome refitting in order to evaluate whether signature adjustment based on nucleotide content improves the results when relying only on a limited subset of mutations.

6.2.3 Explained variance of exposure vectors

To evaluate how well an obtained exposure vector for the set of COSMIC signatures can explain the variance of the somatic mutation patterns of a donor after decomposition (refitting) of the tumor genome, or the respective subset, `decompTumor2Sig` computes the coefficient of determination R^2 [1].

Supplementary Fig. 5 illustrates that the explained variance strongly depends on the number of mutations which are available for signature refitting. When using mutations from promoter regions, region-specific refitting can eventually explain the observed variance in mutation patterns nearly as well as whole-genome refitting (when the promoter regions of a donor contain about 1000 somatic mutations or more; see upper panel of Supplementary Fig. 5).

CTCF binding sites (lower panel of Supplementary Fig. 5), however, are much smaller and usually contain much less somatic mutations from which to estimate exposures. Thus, signature refitting based on the small number of mutations can explain the variance observed in the subset of mutations less well. However, adjusting mutational signatures according the trinucleotide content of the set of CTCF binding sites prior to signature refitting significantly increases the explained variance. The analysis suggests that variances are explained reasonably well for donors with at least 35 somatic mutations located at CTCF binding sites.

6.2.4 Accuracy of exposure vectors

To evaluate the accuracy of exposure vectors obtained from region-specific signature refitting, i.e., the degree to which they approximate those obtained from whole-genome signature refitting, we used two commonly used measures. First,

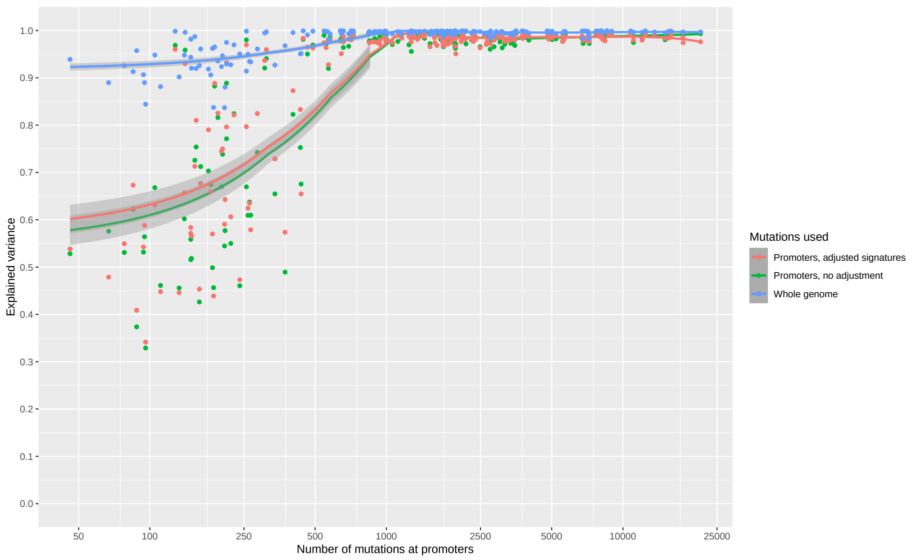

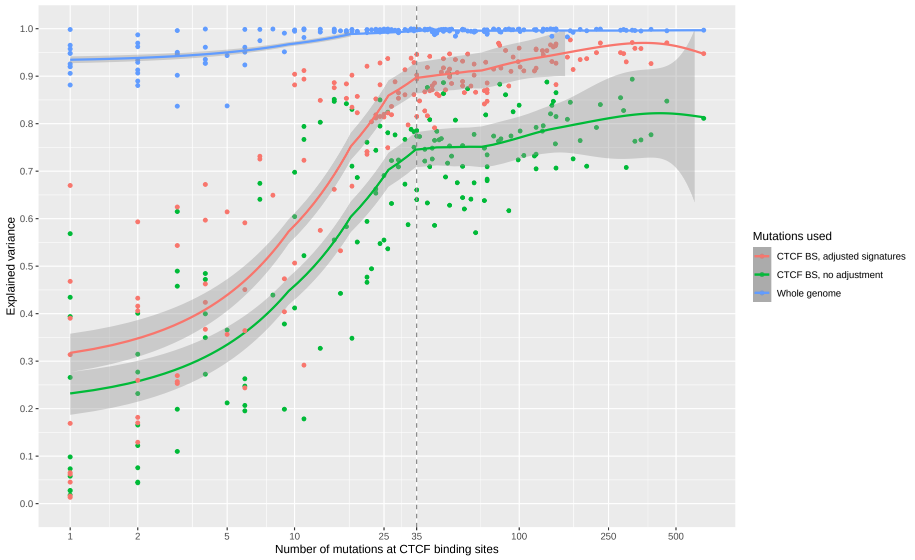

Supplementary Figure 5: Variance (as a function of the number of mutations in the subset of regions) of the tumor genome, or the respective subset, explained by exposure vectors for the three signature refitting approaches for promoter regions (upper panel) and CTCF binding sites (lower panel). The dashed vertical line in the lower panel represents a reasonable cutoff for the number of mutations (n=35) in CTCF binding sites. Dataset: MELA.

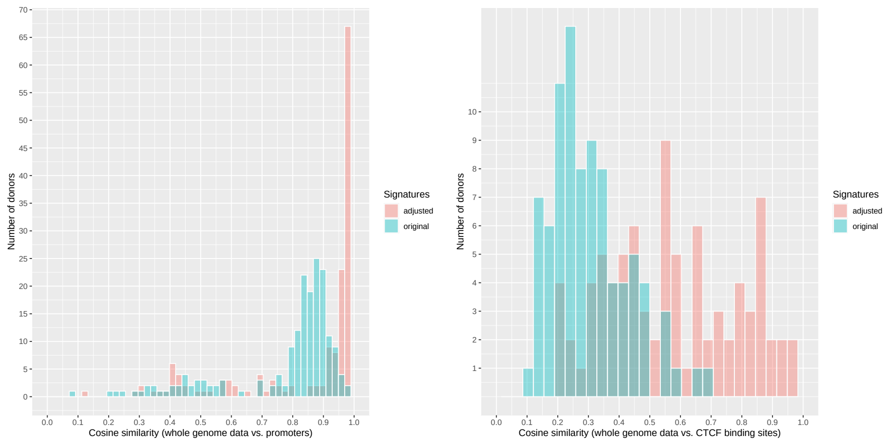

Supplementary Figure 6: Distribution of cosine similarities between exposure vectors obtained from signature refitting for genome-wide mutation data and from signature refitting for mutation data from a limited region set only (with and without prior adjustment of mutational signatures according to trinucleotide frequencies in the specific regions). Left: promoter regions; right: CTCF binding sites, including only donors with at least 35 somatic mutations in CTCF binding sites.

we computed the cosine similarities between the exposure vectors, which ideally approach 1 if the vectors point into the same direction of the n-dimensional space (here: n=number of signatures). Then, we computed the mean squared error (MSE) between the exposure vectors, which ideally approaches 0 if there is little difference between the exposures obtained for the single mutational signatures.

Supplementary Figures 6 and 7 illustrate that cosine similarities between exposures from region-specific refitting and whole-genome refitting are generally improved when mutational signatures are adjusted to region-specific trinucleotide frequencies. How well exposure vectors from whole-genome refitting are approximated depends on the number of mutations available for signature refitting.

For the larger promoter regions, which contain many more mutations than CTCF binding sites in the analyzed melanomas, we can observe strong similarities between exposure vectors from region-specific and whole-genome refitting, especially when signatures are adjusted for trinucleotide content. But also for CTCF binding sites cosine similarities can be significantly improved when signatures are adjusted prior to refitting.

This finding is confirmed when measuring mean squared errors between exposure vectors from region-specific signature refitting and those from whole-genome signature refitting, as illustrated by Supplementary Figures 8 and 9.

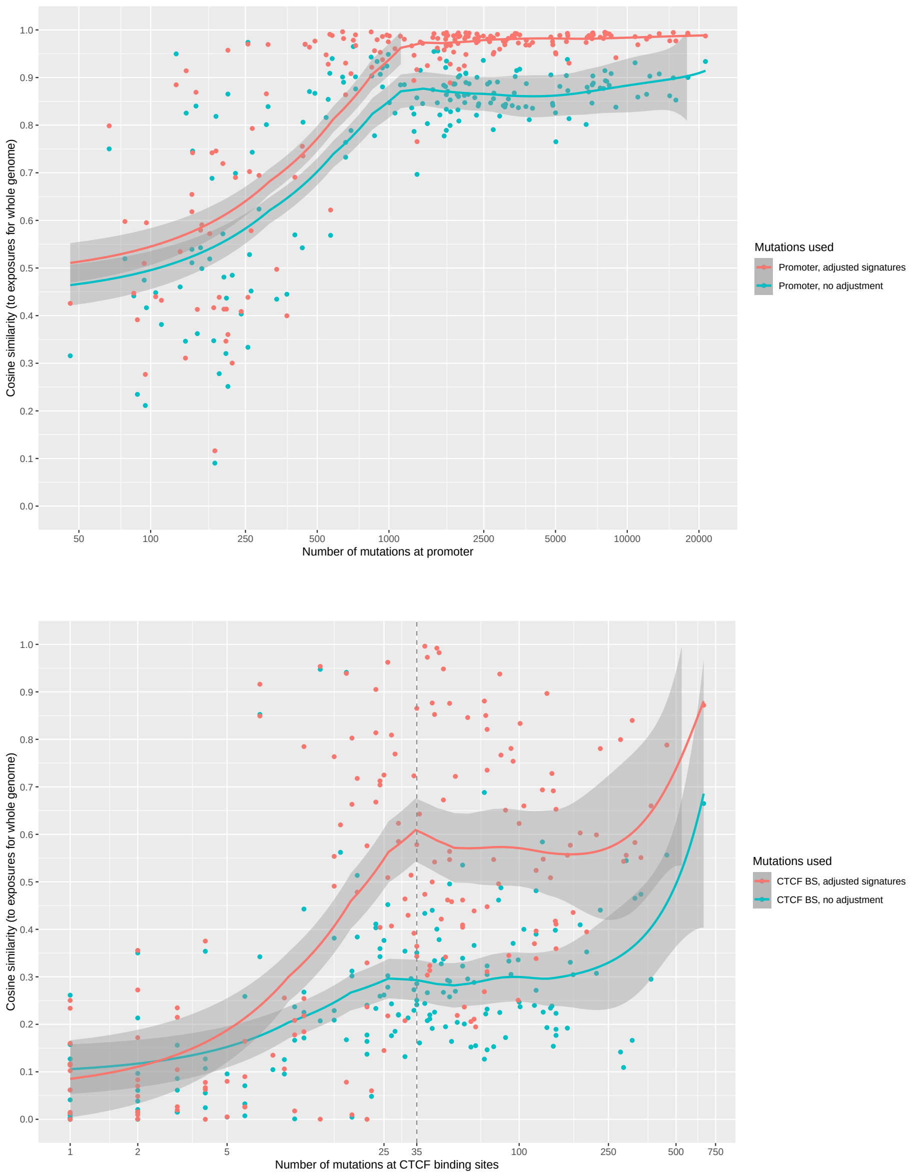

Supplementary Figure 7: Cosine similarity (as a function of the number of mutations) between exposure vectors obtained from signature refitting for genome-wide mutation data and from signature refitting for mutation data from a subset of regions only (with and without prior adjustment of mutational signatures according to trinucleotide frequencies in CTCF binding sites). Upper panel: mutation data from promoter regions; lower panel: mutation data from CTCF binding sites. The dashed vertical line in the lower panel represents a reasonable cutoff for the number of mutations (n=35) in CTCF binding sites (see Supplementary Fig. 5).

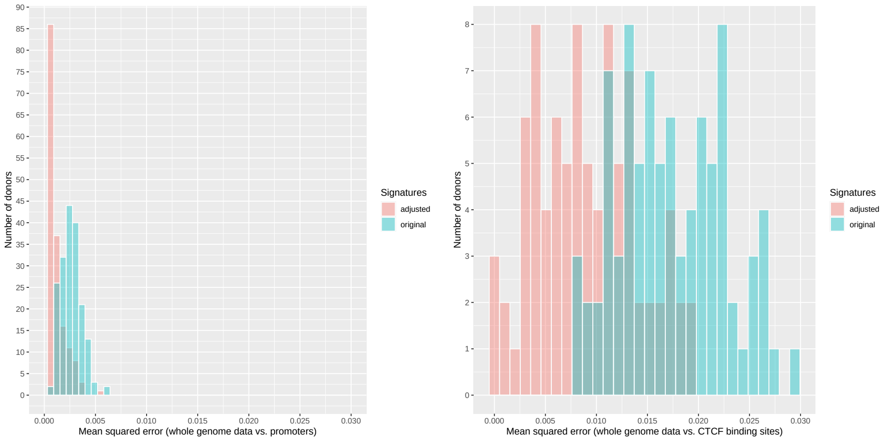

Supplementary Figure 8: Distribution of mean squared errors between exposure vectors obtained from signature refitting for genome-wide mutation data and from signature refitting for mutation data from a limited region set only (with and without prior adjustment of mutational signatures according to trinucleotide frequencies in the specific regions). Left: promoter regions; right: CTCF binding sites, including only donors with at least 35 somatic mutations in CTCF binding sites.

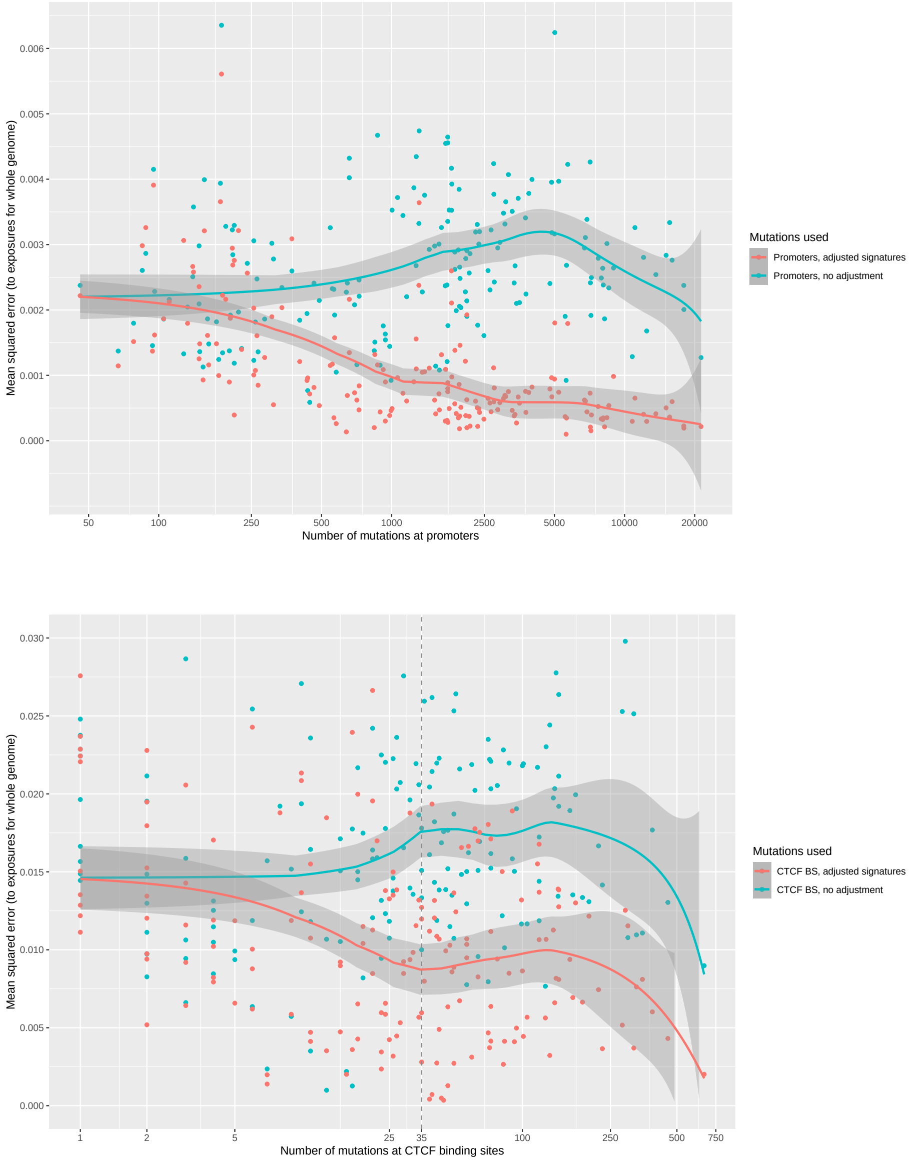

Supplementary Figure 9: Mean squared errors (as a function of the number of mutations) between exposure vectors obtained from signature refitting for genome-wide mutation data and from signature refitting for mutation data from a subset of regions only (with and without prior adjustment of mutational signatures according to trinucleotide frequencies in CTCF binding sites). Upper panel: mutation data from promoter regions; lower panel: mutation data from CTCF binding sites. The dashed vertical line in the lower panel represents a reasonable cutoff for the number of mutations (n=35) in CTCF binding sites (see Supplementary Fig. 5).

7 Example study: CTCF binding site mutations at TAD boundaries

7.1 Introduction: Topologically associated domains and cancer

The human genome is organized hierarchically into discrete topologically associated domains (TADs), self-interacting sub-megabase segments that are relatively insulated from neighboring domains [3, 4] and whose boundaries are associated with particular spatial patterns of CTCF binding sites [5].

Recent studies have shown that TAD disruption is often found in cancer cells and may contribute to oncogenesis through several mechanisms. For instance, the mutation, deletion or epigenetic alteration of a TAD boundary might fuse two adjacent TADs and lead to aberrant gene expression [6, 7, 8, 9]. Other studies have found an enrichment of CTCF binding site mutations at TAD boundaries in various cancer types [9, 10, 11, 12, 13]. A recent review [14] summarizes various findings about the increase of somatic mutation frequencies at transcription factor binding sites, and suggests that this increase is shaped by the complex interplay between DNA damage and the activity of DNA repair mechanisms.

7.1.1 Result: enrichment of CTCF binding site mutations at TAD boundaries

Here, we illustrate that MutViz 2.0 can be used to study the impact of mutational processes on active TAD boundaries and the enrichment of CTCF binding site mutations they generate. Indeed, in particular our own study of the subject [10] has spurred the development of MutViz.

First, we visualized the enrichment of somatic single nucleotide mutations in active CTCF binding motifs at TAD boundaries and compare them with mutations in active CTCF binding motifs which are not located at TAD boundaries. For this distinction, we exploit a study on the H1 human embryonic stem cell line (H1-hESC) which inferred neighborhoods via ChIA-PET (chromatin interaction analysis by paired-end tag sequencing), using the cohesin subunit SMC1 as target [6].

In detail, we identified potential CTCF binding sites using their position frequency matrix (Jaspar 2018 [15]; accession MA0139.1) and Biostrings [16] with a threshold of 80%. We then classified these CTCF binding sites as "in-boundary" or "off-boundary" according to whether they are located at a previously mapped TAD boundary or not, and as "active" or "inactive" according to whether or not they are located at a peak identified in CTCF ChIP-seq data of human H1-hESC cells (produced by the Bernstein Laboratory and published as part of the ENCODE project [17]; accession ENCSR000AMF for H1-hESC).

Supplementary Fig. 10, produced with the MutViz 2.0 histogram view, clearly shows for three different tumor datasets a strong enrichment of mutations at active, in-boundary CTCF binding sites in contrast to also active, but off-boundary CTCF binding sites. Although for both the in-boundary and the off-boundary CTCF sites, the activity (active CTCF binding) in these tumors need not be the same as in H1-hESC, this remarkable result is likely associated with the different biological function of the two sets of binding sites.

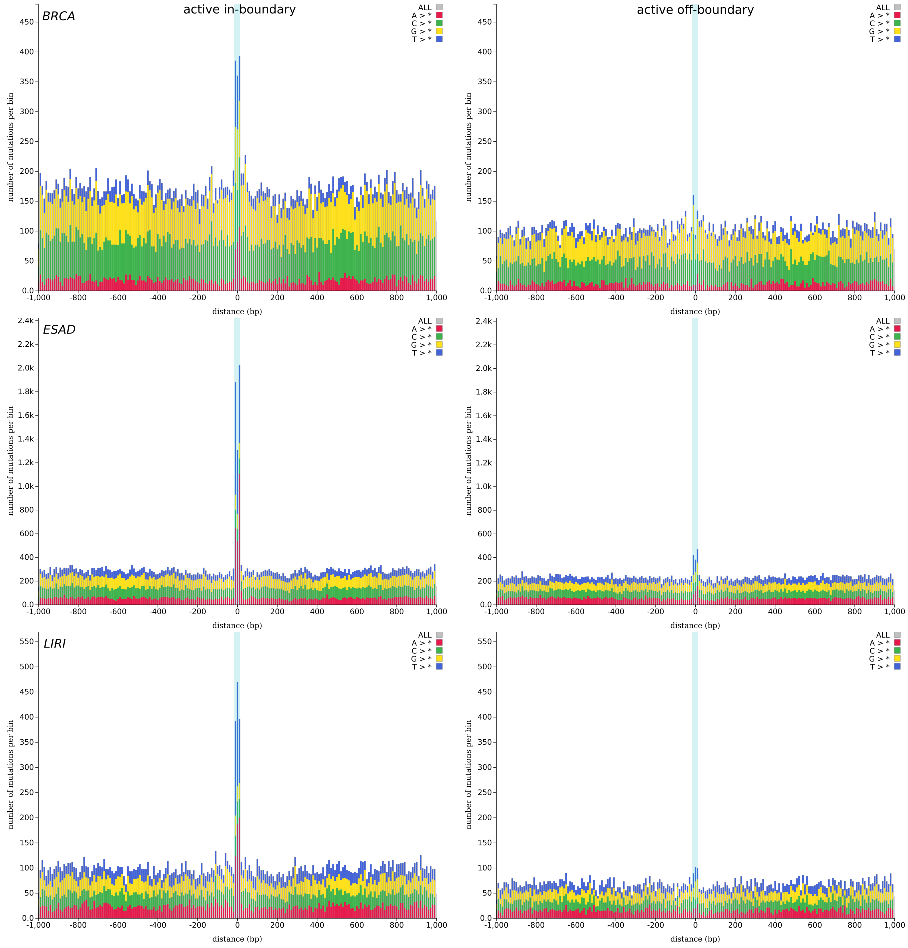

Supplementary Figure 10: Somatic mutation enrichment at the 19bp CTCF binding sites (highlighted by a light blue background) as compared to their flanking genomic regions (-1kbp to +1kbp from the center of each binding site) for both active in-boundary (left) and active off-boundary binding sites (right). Results are shown for three WGS tumor datasets (see Supplementary Table 1): breast cancer (BRCA), esophageal adenocarcinoma (ESAD), and liver cancer (LIRI).

7.2 Result: types of mutations in CTCF binding sites at TAD boundaries in esophageal adenocarcinoma

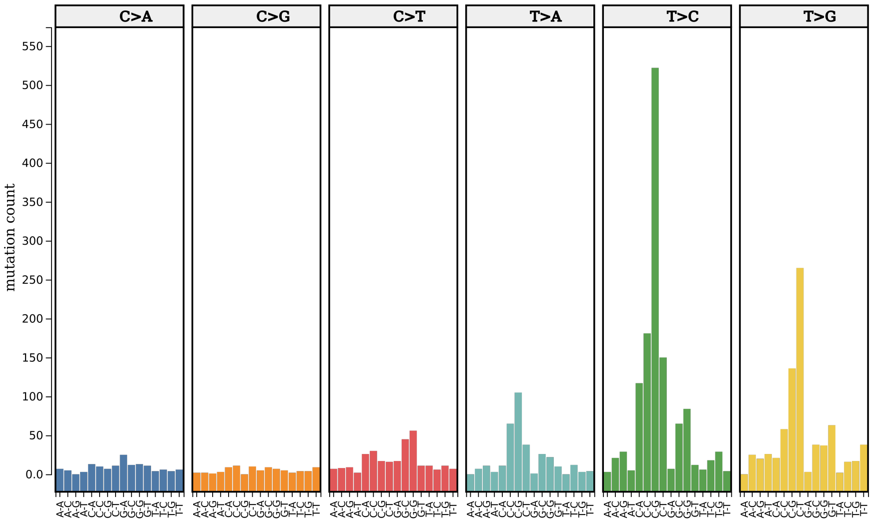

Supplementary Figure 11: Trinucleotide mutation distribution of esophageal adenocarcinoma (ESAD) mutations falling in active in-boundary CTCF binding sites.

The trinucleotide mutation view in Supplementary Fig. 11 illustrates which types of mutations (base changes within the context of their flanking bases) fall in active in-boundary CTCF binding sites in esophageal adenocarcinoma (ESAD). As we can observe, most mutations are T→C and T→G, especially regarding triplets with an upstream cytosine, i.e., C[$T \rightarrow$(C,G)]N, where N can be any nucleotide.

Finally, Supplementary Fig. 12 shows the "mutations per donor" view with the distributions of the number of specific base changes in CTCF binding sites across the individual donors of the ESAD dataset. In addition, we can see a greater number of transversions than transitions.

References

[1] Krüger, S. and Piro, R. M. (2019) decompTumor2Sig: identification of mutational signatures active in individual tumors. *BMC Bioinformatics,* **20**(Suppl 4), 152.

[2] Alexandrov, L. B., Kim, J., Haradhvala, N. J., Huang, M. N., Ng, A. W. T., Wu, Y., Boot, A., Covington, K. R., Gordenin, D. A., Bergstrom, E. N., et al. (2020) The repertoire of mutational signatures in human cancer. *Nature,* **578**(7793), 94–101.

[3] Bickmore, W. A. and van Steensel, B. (2013) Genome architecture: domain organization of interphase chromosomes. *Cell,* **152**(6), 1270–1284.

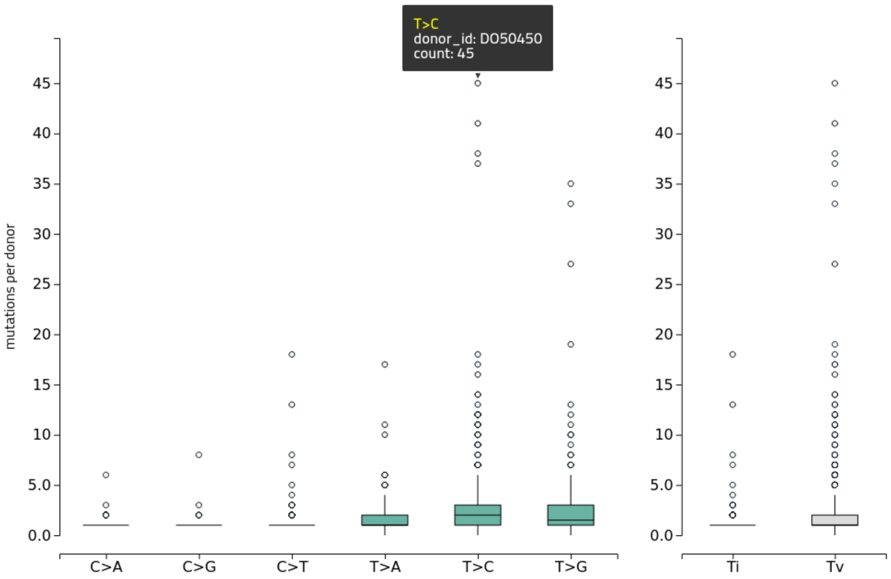

Supplementary Figure 12: Box plot of the number of specific base changes per patient for mutations falling in active in-boundary CTCF binding sites. Outlier details can be visualized, as shown here.

[4] Dixon, J. R., Selvaraj, S., Yue, F., Kim, A., Li, Y., Shen, Y., Hu, M., Liu, J. S., and Ren, B. (2012) Topological domains in mammalian genomes identified by analysis of chromatin interactions. *Nature,* **485**(7398), 376–380.

[5] Nanni, L., Ceri, S., and Logie, C. (2020) Spatial patterns of CTCF sites define the anatomy of TADs and their boundaries. *Genome Biology,* **21**(1), 197.

[6] Ji, X., Dadon, D. B., Powell, B. E., Fan, Z. P., Borges-Rivera, D., Shachar, S., Weintraub, A. S., Hnisz, D., Pegoraro, G., Lee, T. I., et al. (2016) 3D chromosome regulatory landscape of human pluripotent cells. *Cell Stem Cell,* **18**(2), 262–275.

[7] Katainen, R., Dave, K., Pitkänen, E., Palin, K., Kivioja, T., Välimäki, N., Gylfe, A. E., Ristolainen, H., Hänninen, U. A., Cajuso, T., et al. (2015) CTCF/cohesin-binding sites are frequently mutated in cancer. *Nature Genetics,* **47**(7), 818–821.

[8] Flavahan, W. A., Drier, Y., Liau, B. B., Gillespie, S. M., Venteicher, A. S., Stemmer-Rachamimov, A. O., Suvà, M. L., and Bernstein, B. E. (2016) Insulator dysfunction and oncogene activation in IDH mutant gliomas. *Nature,* **529**(7584), 110–114.

[9] Hnisz, D., Weintraub, A. S., Day, D. S., Valton, A.-L., Bak, R. O., Li, C. H., Goldmann, J., Lajoie, B. R., Fan, Z. P., Sigova, A. A., et al. (2016)

Activation of proto-oncogenes by disruption of chromosome neighborhoods. *Science,* **351**(6280), 1454–1458.

[10] Pinoli, P., Stamoulakatou, E., Nguyen, A.-P., Rodríguez Martínez, M., and Ceri, S. (2020) Pan-cancer analysis of somatic mutations and epigenetic alterations in insulated neighbourhood boundaries. *PloS One,* **15**(1), e0227180.

[11] Poulos, R. C., Thoms, J. A., Guan, Y. F., Unnikrishnan, A., Pimanda, J. E., and Wong, J. W. (2016) Functional mutations form at CTCF-cohesin binding sites in melanoma due to uneven nucleotide excision repair across the motif. *Cell Reports,* **17**(11), 2865–2872.

[12] Sabarinathan, R., Mularoni, L., Deu-Pons, J., Gonzalez-Perez, A., and López-Bigas, N. (2016) Nucleotide excision repair is impaired by binding of transcription factors to DNA. *Nature,* **532**(7598), 264–267.

[13] Guo, Y. A., Chang, M. M., Huang, W., Ooi, W. F., Xing, M., Tan, P., and Skanderup, A. J. (2018) Mutation hotspots at CTCF binding sites coupled to chromosomal instability in gastrointestinal cancers. *Nature Communications,* **9**(1), 1–14.

[14] Gonzalez-Perez, A., Sabarinathan, R., and Lopez-Bigas, N. (2019) Local determinants of the mutational landscape of the human genome. *Cell,* **177**(1), 101–114.

[15] Khan, A., Fornes, O., Stigliani, A., Gheorghe, M., Castro-Mondragon, J. A., vanderLee, R., Bessy, A., Chneby, J., Kulkarni, S. R., Tan, G., Baranasic, D., Arenillas, D. J., Sandelin, A., Vandepoele, K., Lenhard, B., Ballester, B., Wasserman, W. W., Parcy, F., and Mathelier, A. (2017) JASPAR 2018: update of the open-access database of transcription factor binding profiles and its web framework. *Nucleic Acids Research,* **46**(D1), D260–D266.

[16] Pagès, H., Aboyoun, P., Gentleman, R., and DebRoy, S. Biostrings: efficient manipulation of biological strings. R package version 2.52.0. (2019).

[17] Dunham, I., Kundaje, A., Aldred, S. F., Collins, P. J., Davis, C. A., et al. (2012) An integrated encyclopedia of DNA elements in the human genome. *Nature,* **489**(7414), 57–74.
